# Deep sympatric mitochondrial divergence without reproductive isolation in the common redstart *Phoenicurus phoenicurus*

**DOI:** 10.1002/ece3.398

**Published:** 2012-11-02

**Authors:** Silje Hogner, Terje Laskemoen, Jan T Lifjeld, Jiri Porkert, Oddmund Kleven, Tamer Albayrak, Bekir Kabasakal, Arild Johnsen

**Affiliations:** 1 National Centre for Biosystematics, Natural History Museum, University of OsloP.O. Box 1172, Blindern, NO-0318, Oslo, Norway; 2 Gocarova542, Hradec Kralove, 500 02, Czech Republic; 3 Norwegian Institute for Nature Research – NINAP.O. Box 5685, Sluppen, 7485, Trondheim, Norway; 4 Department of Biology, Science and Art Faculty, University of Mehmet Akif ErsoyBurdur, Turkey

**Keywords:** Assortative mating, mtDNA, reproductive isolation, sympatric divergence

## Abstract

Mitochondrial DNA usually shows low sequence variation within and high sequence divergence among species, which makes it a useful marker for phylogenetic inference and DNA barcoding. A previous study on the common redstart (*Phoenicurus phoenicurus*) revealed two very different mtDNA haplogroups (5% K2P distance). This divergence is comparable to that among many sister species; however, both haplogroups coexist and interbreed in Europe today. Herein, we describe the phylogeographic pattern of these lineages and test hypotheses for how such high diversity in mtDNA has evolved. We found no evidence for mitochondrial pseudogenes confirming that both haplotypes are of mitochondrial origin. When testing for possible reproductive barriers, we found no evidence for lineage-specific assortative mating and no difference in sperm morphology, indicating that they are not examples of cryptic species, nor likely to reflect the early stages of speciation. A gene tree based on a short fragment of cytochrome c oxidase subunit 1 from the common redstart and 10 other *Phoenicurus* species, showed no introgression from any of the extant congenerics. However, introgression from an extinct congeneric cannot be excluded. Sequences from two nuclear introns did not show a similar differentiation into two distinct groups. Mismatch distributions indicated that the lineages have undergone similar demographic changes. Taken together, these results confirm that deeply divergent mitochondrial lineages can coexist in biological species. Sympatric mtDNA divergences are relatively rare in birds, but the fact that they occur argues against the use of threshold mtDNA divergences in species delineation.

## Introduction

Many species exhibit high levels of intraspecific morphological and genetic variation. Variation in mitochondrial DNA (mtDNA) is particularly prevalent, due to its faster evolutionary rate compared with nuclear DNA (Avise 2000). Usually, such variation is confined to allopatric populations and can be explained by long periods of isolation with differing selection pressures and/or divergence due to genetic drift (Coyne and Orr 2004; Price 2008). High intraspecific mtDNA variation between individuals living in sympatry is less common, and more difficult to explain. Upon closer inspection, such divergent sympatric lineages often show evidence of divergence in other parts of the genome as well as reproductive isolation between the lineages, implying that they are in fact cryptic species (Hebert et al. 2004a; Haine et al. 2006). The concept of DNA barcoding, which applies the mitochondrial cytochrome c oxidase subunit 1 (COI) marker in animals, is based on the premise that there is low variation within species and large divergence gaps between sister species (Hebert et al. 2003, 2004b). Accordingly, provisional species are often suggested when sequence divergence exceeds a certain threshold (e.g., 10 times average intraspecific variation; Carr et al. 2011; Kerr et al. 2007). However, there are examples of sympatric intraspecific divergences of a magnitude that exceeds normal sister species-level divergence (Wayne et al. 1990; Tominaga et al. 2009; Xiao et al. 2012), making species discrimination based on a strict divergence threshold in mtDNA too simplistic (Moritz and Cicero 2004).

In birds, deep sympatric mtDNA divergences have been found in a few species (e.g., Quinn 1992; Webb et al. 2011; Kerr et al. 2009b; Johnsen et al. 2010; Barrowclough et al. 2011). For example, common ravens (*Corvus corax*) show a 4% divergence between Holarctic and western North American lineages, with a high degree of sympatry and interbreeding (Webb et al. 2011) and males of the (*Manacus manacus*) collected from a single lek represented two groups with 3.5% divergence (Kerr et al. 2009b). The interpretation of such deep sympatric divergences is challenging and requires additional information about potential methodological pitfalls in mtDNA sequencing and reproductive barriers to gene flow in nuclear DNA.

Several hypotheses have been proposed to explain such high mtDNA variation (Webb et al. 2011). First, sympatric intraspecific divergences in mtDNA may be an artifact caused by nuclear mitochondrial pseudogenes, or numts. This is genetic material that has been translocated from the mitochondrial to the nuclear genome. These copies are assumed to be nonfunctional (Bensasson et al. 2001), evolve fast, accumulate frame shifts and stop codons, and show double peaks when sequenced (Bertheau et al. 2011; Triant and Hayes 2011). Second, as stated above, the divergent lineages may in fact reflect cryptic species, implying that the taxonomy is incorrect. Recent avian examples include thrushes (*Turdus spp*.) in western Amazonas (O'Neill et al. 2011), the winter wren (*Troglodytes troglodytes*) in North America (Toews and Irwin 2008), and seven nonpasserine migratory birds from the Philippines (Lohman et al. 2010).

A third possibility is that high mtDNA variation is caused by hybridization with a closely related species, which can lead to introgression of mtDNA (Coyne and Orr 2004; Bachtrog et al. 2006; Toews & Brelsford, 2012). Hybridization is common in birds, occurring in approximately one out of 10 species (Grant and Grant 1992; McCarthy 2006). However, as females are the heterogametic sex in birds, female hybrids are more likely to be affected by reduced viability and/or fertility than males (Haldane 1922), which reduces the likelihood of introgression of the maternally inherited mtDNA. Nevertheless, Taylor et al. (2011) found evidence for hybridization between the sister species common murre (*Uria aalge*) and thick-billed murre (*U. lomvia*), with mtDNA introgression from the thick-billed murre into the common murre. Another example is mtDNA introgression between the golden-winged warblers (*Vermivora chrysoptera*) and the blue-winged warblers (*V. cyanoptera*) in North America (Shapiro et al. 2004).

Fourth, deep mtDNA divergence can reflect long periods of geographical isolation followed by secondary contact. The divergence might be a result of neutral differences within a single species, and thus represent a historical artifact of divergent lineages that have remerged (Webb et al. 2011). In the absence of reproductive barriers, such remerging lineages will be expected to collapse into one (speciation in reverse). The fixation of ancestral allelic lineages can be due to either drift or selection, and it produces a reciprocally monophyletic gene tree (Neigel and Avise 1986). If the two populations have been separated long enough, with little or no gene flow between them, they may have accumulated genetic and phenotypic differences, which might result in reproductive barriers in the form of different morphological, physiological or behavioral traits (Coyne and Orr 2004). Reproductively isolated forms might thus arise if local adaptations are strong, colonization of alternative habitats is eliminated and reproductive contact is reduced (Nosil et al. 2005; Sobel et al. 2010). If secondary contact later occurs, a shift in mate recognition systems and mate preferences may lead to assortative mating (precopulatory barrier) or gamete incompatibilities (postcopulatory, prezygotic barrier) as a result of the earlier allopatry, and the genetic variation between the two populations will be maintained (early speciation). Sperm morphology has a genetic basis (Birkhead et al. 2005), shows remarkable levels of diversification (reviewed in Pitnick et al. 2009) and has shown geographical variation in some avian studies (Lüpold et al. 2011; Schmoll and Kleven 2011). Differences in sperm morphology may thus contribute to prezygotic reproductive barriers in the early stages of speciation (Coyne and Orr 2004). Finally, deep mtDNA divergence may reflect maintenance of two or more ancestral lineages in a panmictic population with large effective population size (Avise et al. 1988; Webb et al. 2011).

In a recent DNA barcoding study, Johnsen et al. (2010) found two different COI lineages in the common redstart. The divergence between these two haplotype lineages (hereafter referred to as haplogroups) was in the magnitude of 5%, suggesting that these lineages separated about 2 million years ago according to the conventional molecular clock estimate (Bromham and Penny 2003; but see Pulquério and Nichols 2007; Weir and Schluter 2008). These two haplogroups were initially found to interbreed in one mixed pair from Norway (Johnsen et al. 2010). Our main aims in the present study are twofold. First, we describe the distribution of the two haplogroups found in Johnsen et al. (2010) across the breeding range of the common redstart, and hence examine their degree of sympatry and interbreeding in detail. Second, we explore the above hypotheses for how this deep mtDNA variation may have originated, combining sequence data from two mtDNA regions (control region and COI) and two nuclear Z-linked introns (BRM-15 and ALDOB-6), with data on degree of assortative mating and sperm size variation between the haplogroups. We test the following predictions related to each hypothesis. (1) From the numt hypothesis, we predict to find stop codons and double peaks in the sequences. (2) From the cryptic species hypothesis, we predict to find reproductive barriers, such as assortative mating or differences in the sperm morphology, and divergence in nuclear DNA that is related to the divergence in mtDNA. (3) From the recent hybridization hypothesis, we predict that one of the haplogroups would cluster together with one of the other extant *Phoenicurus* species. (4) From the geographic isolation hypothesis, we predict that there will be structure in the geographical distribution of the two haplogroups, and that they will show different mismatch distributions due to different demographic histories. If the lineages are in the process of remerging (speciation in reverse), there should be no reproductive barriers and little or no structure in the nuclear sequences with respect to the mtDNA lineages yet high nuclear nucleotide variation, whereas if they are in the process of further divergence (early speciation), we would predict to find some evidence for reproductive isolation and a pattern of divergence in the nuclear data as a result of the two lineages being effectively separated. Finally, (5) from the coexistence in one panmictic population hypothesis, we predict lack of geographic structure and reproductive barriers, similar mismatch distributions, and no divergence in the nuclear introns.

## Materials and Methods

### Study species

**Figure 1:**
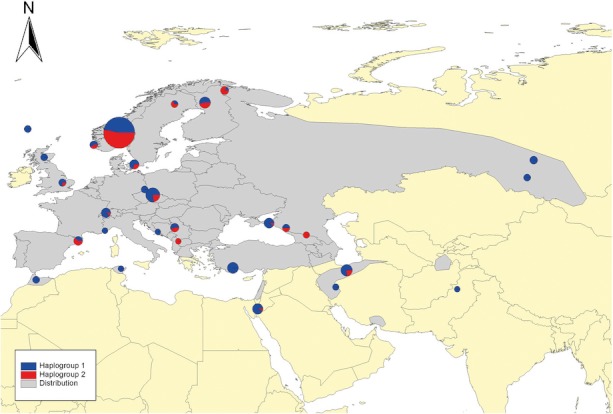
Map showing the distribution of the common redstart (shaded area), and the sampling locations (circles) with the relative frequency of the two haplogroups (blue = haplogroup 1 and red = haplogroup 2). Breeding birds are collected from all locations, except from Israel (migrating birds). On the basis of all common redstart samples (*N* = 387), both sequenced for the long COI and the short COI fragment.

The common redstart is a small (∼15 g), sexually dimorphic passerine bird, breeding in the Western Palearctic (Fig. 1), and wintering in North Africa. The breeding system is predominantly social and genetic monogamy (Kleven et al. 2007), but instances of polygyny have been observed (del Hoyo et al. 2005).

### Samples

#### High quality DNA

Breeding redstarts from Norway, the Czech Republic, Finland, Morocco, Mongolia, Iran, Spain, and Turkey were caught at their respective breeding grounds during spring 2002, 2006, 2009, 2010, and 2012. We collected up to 25-μl blood by brachial venipuncture and stored the blood in 96% ethanol. In addition, blood samples were collected from migratory birds from Israel (see Table 1 for sample details). Birds were caught using mist nets and playback in the beginning of their breeding season in their breeding territories, or with a clap-net (with a meal-worm for a bait) during the period of feeding the chicks. All birds were putatively unrelated, predominantly adult breeders (*N* = 254), but in some cases (*N* = 22) where the female could not be sampled, we instead sampled one chick per nest as a representative of the female mtDNA lineage. In addition, tissue samples collected from breeding birds in Russia (2004–2006, *N* = 16) and Serbia (2008, *N* = 7) were provided by Yale Peabody Museum (New Heaven) and tissue samples of one Moussier's redstart (*P. moussieri*), one blue-fronted redstart (*P. frontalis*), and two Eversmann's redstart (*P. erythronotus*), were provided from the Natural History Museum of Copenhagen (see SI Table 1 for more information).

**Table 1:** Basic sample information for (a) contemporary common redstarts, where DNA was extracted from either fresh blood, tissue or feather samples and subjected to long COI sequencing, and (b) up to 150-years old museum skins (and one contemporary population from Finland) where DNA was extracted from foot pads or dried blood samples (Finland) and subjected to short COI sequencing

Locality	Subspecies	Latitude	Longitude	*n* m/f/u*	*N* Sperm	Haplotype 1/2	Year
a)							
Norway, south-east	*phoenicurus*	61°16′N	12°17′E	100/78/19	67	94/103	2002–2010
Norway, west	*phoenicurus*	59°18′N	04°52′E	2/2/0	0	2/2	2005
Czech Republic	*phoenicurus*	50°11′N	15°55′E	13/9/9	8	24/7	1991–2010
Russia, Adygea	*phoenicurus*	44°11′N	40°04′E	3/1/0	0	2/2	2004
Russia, Krasnodarskiy Kray	*phoenicurus*	45°04′N	36°59′E	6/5/0	0	11/1	2004–2006
Serbia	*phoenicurus*	44°16′N	19°53′E	0/0/7	0	4/3	2008
Spain	*phoenicurus*	41°50′N	02°23′E	7/1/0	0	3/5	2012
Turkey	*samamisicus*	36°58′N	30°26′E	1/0/15	0	16/0	2010
Iran	*samamisicus*	36°32′N	51°3′E	14/4/0	0	13/5	2010
Morocco	*phoenicurus*	33°32′N	5° 6′W	2/0/0	0	2/0	2008
Israel	*samamisicus*	29º33′N	34º56′E	0/0/14	0	13/1	2007–2008
b)							
Norway, north	*phoenicurus*	69°4′N	28°55′E	0/6/0	0	1/5	1866–1966
Finland	*phoenicurus*	66°54′N	25°22′E	0/17/0	0	9/8	1992–2006
Sweden	*phoenicurus*	66°38′N	19°51′E	3/0/0	0	1/2	1915–1970
Faroe Islands	*phoenicurus*	62°00′N	40°24′W	3/0/0	0	3/0	1898–1910
Norway, south-east	*phoenicurus*	61°16′N	12°17′E	2/0/3	0	4/1	1882–1908
Norway, west	*phoenicurus*	58°41′N	05°34′E	0/1/0	0	1/0	1908
Scotland	*phoenicurus*	57°03′N	3°03′W	3/0/0	0	3/0	1915–1919
Denmark	*phoenicurus*	55°42′N	12°34′E	10/0/0	0	7/3	1890–1977
England	*phoenicurus*	51°46′N	40°10′W	4/0/0	0	3/1	1933–1956
Germany	*phoenicurus*	51°10′N	14°26′E	3/0/0	0	3/0	1893–1952
Switzerland	*phoenicurus*	46°56′N	7°26′E	10/0/0	0	9/1	1930–1953
France	*phoenicurus*	43°42′N	7°14′E	1/0/0	0	1/0	1937
Karaku, Pakistan	*samamisicus*	33°6′N	71°5′E	0/1/0	0	1/0	1876
Croatia	*phoenicurus*	43°30′N	16°55′E	1/0/0	0	1/0	unknown
Russia, Barnaul	*phoenicurus*	53°19′N	83°46′E	3/0/0	0	3/0	1896–1933
Russia, Caucasus	*phoenicurus*	42°55′N	43°45′E	2/0/0	0	0/2	unknown
Russia, Tomsk	*phoenicurus*	56°28′N	84°57′E	5/0/0	0	5/0	1896–1923
Macedonia	*phoenicurus*	41°47′N	20°32′E	1/0/0	0	0/1	1935
Turkey	*samamisicus*	36°58′N	30°26′E	1/0/0	0	1/0	1876
Tunisia	*phoenicurus*	36°49′N	10°09′E	1/0/0	0	1/0	1938
Iran	*samamisicus*	36°32′N	51°3′E	0/0/5	0	3/2	2010
Morocco	*phoenicurus*	33°32′N	5°6′W	1/0/0	0	1/0	1919

* m, males; f, females; u, unknown.

#### Degraded DNA

Feather samples were collected from nine females in Finland in June 1992–1994 and blood samples were collected (and dried) from nine unrelated chicks during spring 1998 and 1999 in Hradec Kràlové, Czech Republic. Toe-pad samples were collected from 88 adult museum specimens (*P. phoenicurus*) from the Natural History Museum of London (Tring), Harrison Museum (London), the Natural History Museum of Oslo, and the Natural History Museum of Copenhagen. Of these, the majorities were adult breeders, except three individuals collected in Copenhagen, Denmark and three individuals sampled in the Faroe Islands during migration (SI table 2). Finally, toe-pad samples were collected from Prezevalski's redstart (*P. alaschanicus, N* = 2), blue-capped redstart (*P. caeruleocephalus, N* = 2), blue-fronted redstart (*N* = 2), Hodgson's redstart (*P. hodgsoni, N* = 2), Moussier's redstart (*N* = 2), and white-tailed redstart (*P. schisticeps*, *N* = 2) in order to investigate the relationship between the two *P. phoenicurus* haplogroups and closely related species of the genus *Phoenicurus* (samples were collected from the Natural History Museum of London (Tring), Yale Peabody Museum (New Heaven), Natural History Museum of Copenhagen and Natural History Museum of Oslo. SI Table 2).

#### Genbank sequences

COI sequences from six black redstart (*P. ochruros*)*,* five Eversmann's redstart*,* three Güldenstädt's redstart (*P. erythrogastrus*), and five daurian redstart (*P. auroreus*) were downloaded from Genbank (see SI Table 3 for more information).

### DNA extraction and PCR

DNA from the blood samples was extracted following the protocol for the E.Z.N.A blood kit (Omega Bio-Tek, Inc, Norcross, Georgia). DNA from the toe-pads was extracted following the protocol form E.Z.N.A-tissue Kit (Omega Bio-Tek, Inc, Norcross, Georgia), or using a Mole-extraction robot following the manufacturers' protocol (Mole Genetics AS, Norway).

For the common redstart, two fragments of mtDNA (COI, 700bp and control region, 421bp), and two Z-linked introns (BRM-15, 311bp, and ALDOB-6, 531bp) were sequenced (Genbank accession number JX945383-JX945521). COI was sequenced for a total of 201 redstarts from different populations (Table 1: details and sequences also available at the BOLD website (http://www.barcodinglife.com/), project NorBOL – Birds – Phoenicurus). For the samples consisting of degraded DNA and some of the high quality DNA samples, a short piece of the COI (120bp) was amplified and a restriction enzyme (Aci II) was used to determine the haplogroup (*N* = 88 for degraded DNA and *N* = 98 for high quality DNA). The restriction enzyme was chosen so that it would cut in one of the conserved sites in haplogroup 2, making two bands visible on an electrophoresis gel for this haplogroup. For the other haplogroup, only one band was visible. Ten individuals were also analyzed using both restriction cutting and sequencing in order to confirm the validity of the methods. In order to get the exact haplotype for the Z-linked introns, a total of 54 females of the common redstart were chosen from the south-east population in Norway (of these, only 42 worked for both introns chosen herein). The same 54 individuals were also sequenced for the control region. For the other *Phoenicurus* samples, the long COI (*N* = 4) or short COI (*N* = 12) fragment was sequenced (project NorBOL – Birds – Phoenicurus). All regions were amplified in PCR reaction volumes of 10-μL, containing dH_2_O, 1X PCR buffer II (Applied Biosystems, Foster City, California), 1.5-mM magnesium, 0.2-mM dNTP (ABgene, Epsom, UK), 0.5-mM forward and reverse primer, 3% Dimethyl sulfoxide (DMSO), 0.25 U AmpliTaq DNA polymerase (Applied Biosystems), and approximately 50-ng DNA template. The amplifications were run on a DNA Engine Tetrad 2 (MJ Research, Waterton, MA, USA). The following profile was used: 95°C for 1 min, 94°C for 30 sec, primer-specific annealing temperature (see Table 2) (55–60°C) for 30 sec, 72°C for 1 min, then the second to forth step another 34–39 cycles before the last step, 72°C for 10 min. A 3-μl PCR-product was electrophoresed in 1% agarose TBE to confirm amplification success and to exclude any contamination.

**Table 2:** Primer information and amplification conditions

Locus	Class^1^	Primer sequence (5′-3′)	PCR^2^	Reference
Aldob-6	Z	F: AGACCATGATCTCCAGCGCT	56	Borge et al. 2005
		R: CCTTCCAGGTAGACATGATG		
Brm-15	Z	F: AGCACCTTTGAACAGTGGTT	56	Borge et al. 2005
		R: TACTTTATGGAGACGACGGA		
COI-ExtF	m	F: ACGCTTTAACACTCAGCCATCTTACC	55	Johnsen et al. 2010
BirdR2		R: ACTACATGTGAGATGATTCCGAATCCAG		
PhSa-F1	m	F: AACGTAGTCGTCACAGCCCATGCTT	55	This study
PhSa-R1		R: TTATTCGRGGRAATGCTATG		
L437	m	F: CTCACGAGAACCGAGCTACT	52	Tarr 1995
H1248		R: CATCTTCAGTGTCATGCT		

1 DNA class: Z, Z-linked; m, mtDNA.

2 Annealing temperature.

The remaining PCR-product was purified by digesting unincorporated nucleotides and primers using diluted (1:9) ExoSap-It (United States Biochemical, Cleveland, Ohio) run at 37°C for 45 min followed by 80°C for 15 min to inactivate the enzyme. The PCR products were then sequenced using BigDye Terminator sequencing buffer and v 3.1 Cycle Sequencing kit (Applied Biosystems). The sequences were aligned and edited using ClustalW in the program Mega v 5 (Tamura et al. 2007) and modified manually. Each base was called, using both forward and reverse sequencing reads for each strand. All sequences for each locus were adjusted to the same length as the shortest sequence of that locus for comparison.

### Genetic analyses

Molecular gene trees were constructed using the neighbor-joining method implemented in Mega v 5 (Tamura et al. 2007), using the Kimura two-parameter model and 10,000 bootstrap replicates.

In order to examine the genetic structure of the redstart populations, analysis of molecular variance (AMOVA) was run using the program Arlequin v 3.5 (Excoffier et al. 2005). Pairwise population differences were estimated using the *F*_ST_ statistic (Weir and Cockerham 1984) implemented in Arlequin, with default settings for the population comparisons. In these analyses, we included seven populations with minimum seven individuals sequenced for the long COI fragment. Sequential Bonferroni correction was applied to adjust critical *P*-values for multiple statistical testing (Rice 1989). DNAsp v 5 (Librado and Rozas 2009) was used to calculate nucleotide variation, *π*, of the Z-introns (Hudson et al. 1987).

To test for historical demographic events within the two haplogroups (Johnsen et al. 2010), we first calculated Tajima's *D* (Tajima 1989) for the concatenated COI and control region sequences using DNAsp v 5. The sign of the test statistic can indicate a recent bottleneck (positive Tajima's *D*) or population expansion (negative Tajima's *D*). Second, we compared the observed frequency distribution of pairwise nucleotide differences among individuals within each of the haplogroups with the expected distribution from a sudden population expansion (mismatch distribution), using Arlequin v 3.5. If a population has experienced a long lasting demographic equilibrium or a decline, then a multimodal distribution should be displayed, whereas a unimodal distribution should be displayed if a population has experienced a sudden demographic expansion (Slatkin and Hudson 1991; Rogers and Harpending 1992). However, recent changes are not always detectable in a mismatch distribution, because they might be masked by threshold effects, time lags or earlier demographic events (Rogers and Harpending 1992; Lavery et al. 1996). Arlequin tests the goodness-of-fit to this model using SSD test statistics (the sum of squared differences between the observed and the estimated mismatch distributions; Rogers and Harpending 1992).

We used the general nonlinear least-square approach to estimate the demographic mutation time parameter tau, *τ* = 2*μt*, where *μ* is the mutation rate per generation of the DNA fragment and *t* is the number of generations. Assuming a generation time of 1 year and the standard molecular clock of mtDNA divergence of 2% per million years (Bromham and Penny 2003; but see Weir and Schluter 2008; Lande et al. 2003), we estimated the time since expansion of the two haplogroups using the formula above (see Sætre et al. 2012 for further details).

### Assortative mating

Generalized linear models (GLZ) with binomial distribution were performed in Statistica, to test for assortative mating between the two haplogroups among breeding pairs. We had data from 68 pairs, from Norway (*N* = 60) and Czech Republic (*N* = 8), respectively. However, as there were no haplogroup two females represented in the Czech population, testing for assortative mating would be noninformative and thus this population was excluded.

### Sperm measurements

We obtained sperm samples from 67 males from three subpopulations in south-east Norway (Røros N 62º 37′, E 11º 38′, Trysil N 61º16′, E 12º 17′ and Aurdal N 60º 39′, E 9º37′) and 8 males from the Czech Republic (Hradec Králové N 50º 11′, E 15º 55′). Sperm samples were collected by gently massaging the cloacal protuberance of breeding males using a similar technique as described in Wolfson (1952). The ejaculate was collected using a microcapillary tube and fixed in a 5% formalin solution. Sperm morphology data were obtained for each individual, from 10 normal and undamaged sperm, as 10 sperm provides an accurate estimate of each individual's sperm length (Laskemoen et al. 2007). The following measurements were obtained (±0.1 μm); head length, midpiece length, tail length, flagellum length and total length, where flagellum length is the sum of midpiece + tail length, and total length the sum of head + midpiece + tail. For each sperm trait, we used the means within individuals. All measurements were obtained from digital images captured at a magnification of 160× using a Leica DFC420 camera mounted on a Leica DM6000 B digital light microscope (Leica Microsystems, Switzerland). To avoid observer effects, one person (T.L.) conducted all sperm measurements. All sperm components were normally distributed (Shapiro–Wilks tests, all *W* > 0.96, all *P* > 0.05). Statistical analyses of assortative mating and differentiation in sperm morphology were conducted using Statistica v 7.1 (StatSoft Inc).

## Results

### Haplogroup characterization and distribution

**Figure 2:**
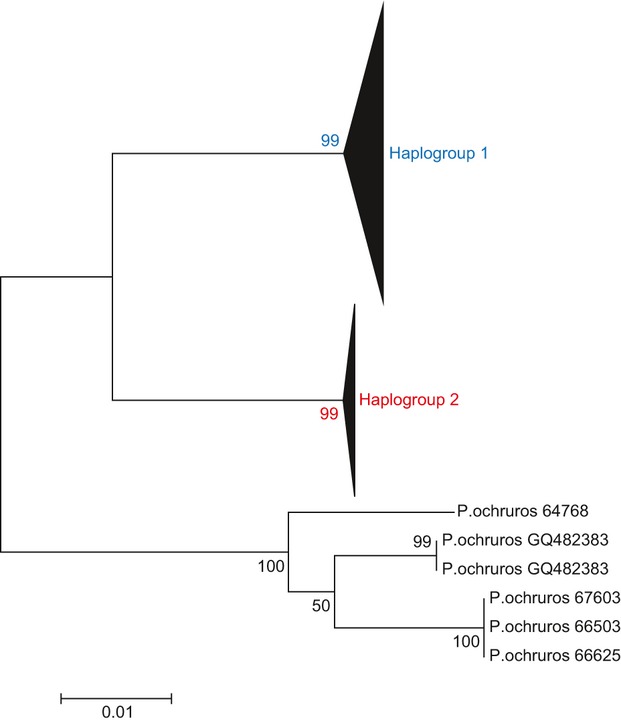
Neighbor-joining tree (K2P, 10,000 bootstrap replicates) based on COI (545bp) for contemporary common redstarts (*N* = 201), with black redstart as outgroup. The two common redstart haplogroups consist of 122 and 79 individuals, respectively. Only bootstrap values above 50% are shown. Blue = haplogroup 1 and red = haplogroup 2.

**Figure 3:**
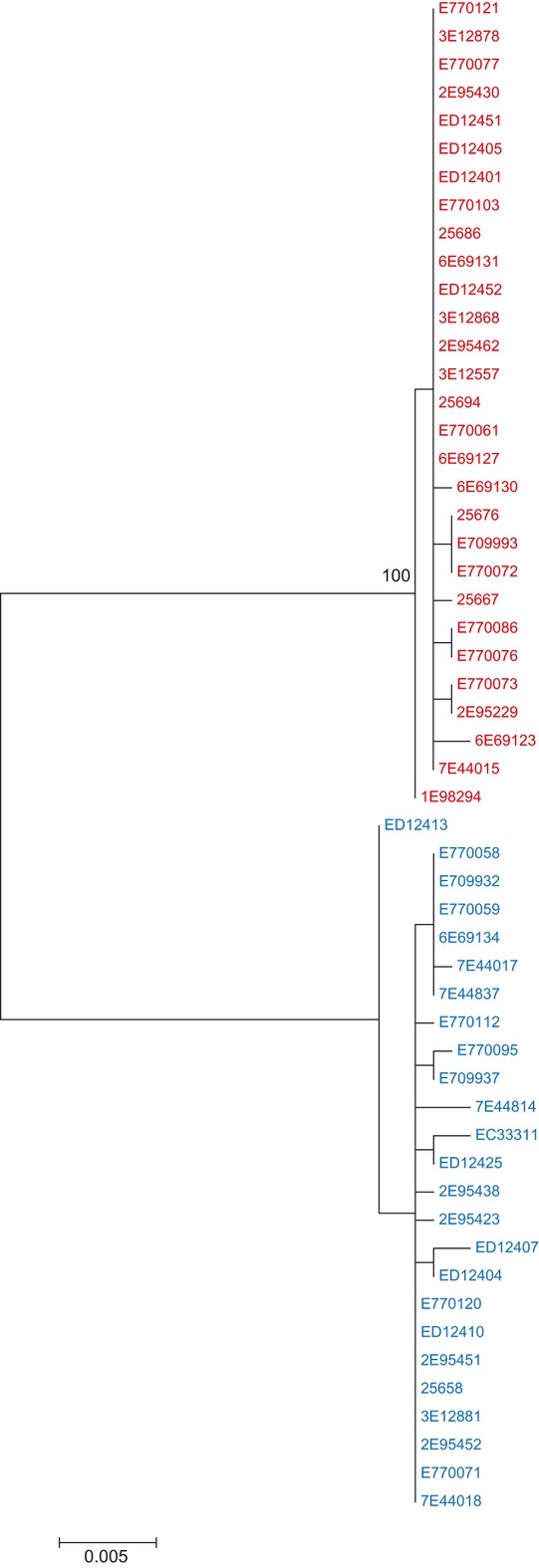
Neighbor-joining tree (K2P, 10,000 bootstrap replicates) based on the COI and control region (1121bp) combined for 54 common redstart females from the south-east Norwegian population. Only bootstrap values above 50% are shown. Blue = haplogroup 1 and red = haplogroup 2.

A gene tree based on the COI region from different populations (Norway, Czech Republic, Iran, Turkey, Morocco, Russia, Spain, and Serbia) with black redstart as an outgroup showed that the COI region consists of two clearly separated groups (Fig. 2), supported by high bootstrap values (98% and 99%). There is some variation within each of these two groups (SI Fig. 1). The split (∼5%) between these two haplogroups suggests that the haplogroups separated about 2 million years ago, assuming a standard avian molecular clock of 2% sequence divergence per million years (e.g., Päckert et al. 2007). We also found two haplogroups for the control region (using the south-east Norwegian population, data not shown), which matched respective groups in the COI as would be expected for two regions in the mt genome. A neighbor-joining tree based on concatenated sequences of the two mtDNA regions is shown in Figure 3.

The two clades coexist in Scandinavia, Great Britain, and central to eastern Europe (Fig. 1). There is an overrepresentation of haplogroup 1 in western Europe and southern parts of the breeding range, whereas haplogroup 2 is mainly located in north and eastern Europe, and usually in coexistence with haplogroup 1. The AMOVA revealed significant differentiation in COI among seven contemporary breeding populations (*F*_ST_ = 0.17, *P* < 0.001). Pairwise comparisons showed significant differentiation between four of the populations (Norway vs. Czech R., and Turkey vs. Norway, Czech R. and Spain; Table 3).

**Table 3:** Pairwise *F*_st_ (below diagonal) with *P*-values (above diagonal), for contemporary breeding populations. Bold = significant after sequential Bonferroni correction

	Norway	Czech Republic	Serbia	Russia	Iran	Turkey	Spain
Norway		**<0.001**	0.77	0.03	0.02	**<0.001**	0.48
Czech Republic	0.22		0.12	0.38	0.09	**<0.001**	0.01
Serbia	−0.09	0.13		0.58	0.34	0.01	0.36
Russia	0.11	0.00	−0.06		0.43	0.01	0.03
Iran	0.10	0.10	−0.07	−0.03		0.02	0.03
Turkey	0.33	0.17	0.45	0.14	0.16		**<0.001**
Spain	−0.03	0.41	−0.07	0.21	0.17	0.63	

**Figure 4:**
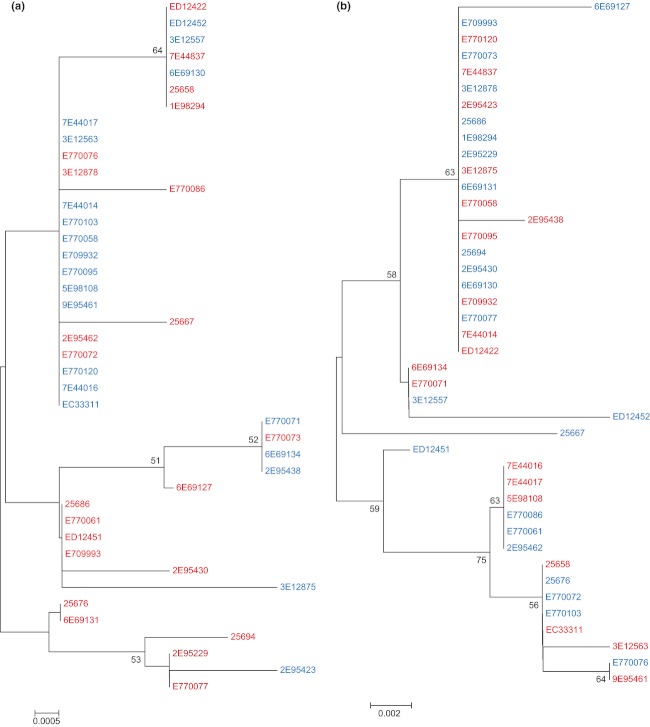
Neighbor-joining tree (K2P, 10,000 bootstrap replicates) based on two nuclear introns (a) = ALDOB-6 and (b) = BRM-15 for 42 common redstart females from the Norwegian south east population. Only bootstrap values above 50% are shown. Blue = haplogroup 1 and red = haplogroup 2.

When analyzing Z-intron variation among 42 females from the Norwegian population, we found no distinct groups related to the ones found in the mtDNA analyses (Fig. 4a,b). This is expected, as the Z-introns and mtDNA would segregate independently within a single population. We found the combined nucleotide diversity, the *π*-value, for the two introns to be 0.00614 (± 0.00043), which is the second highest *π*-value among 22 passerines sequenced in our lab (0.00079–0.00621).

**Figure 5:**
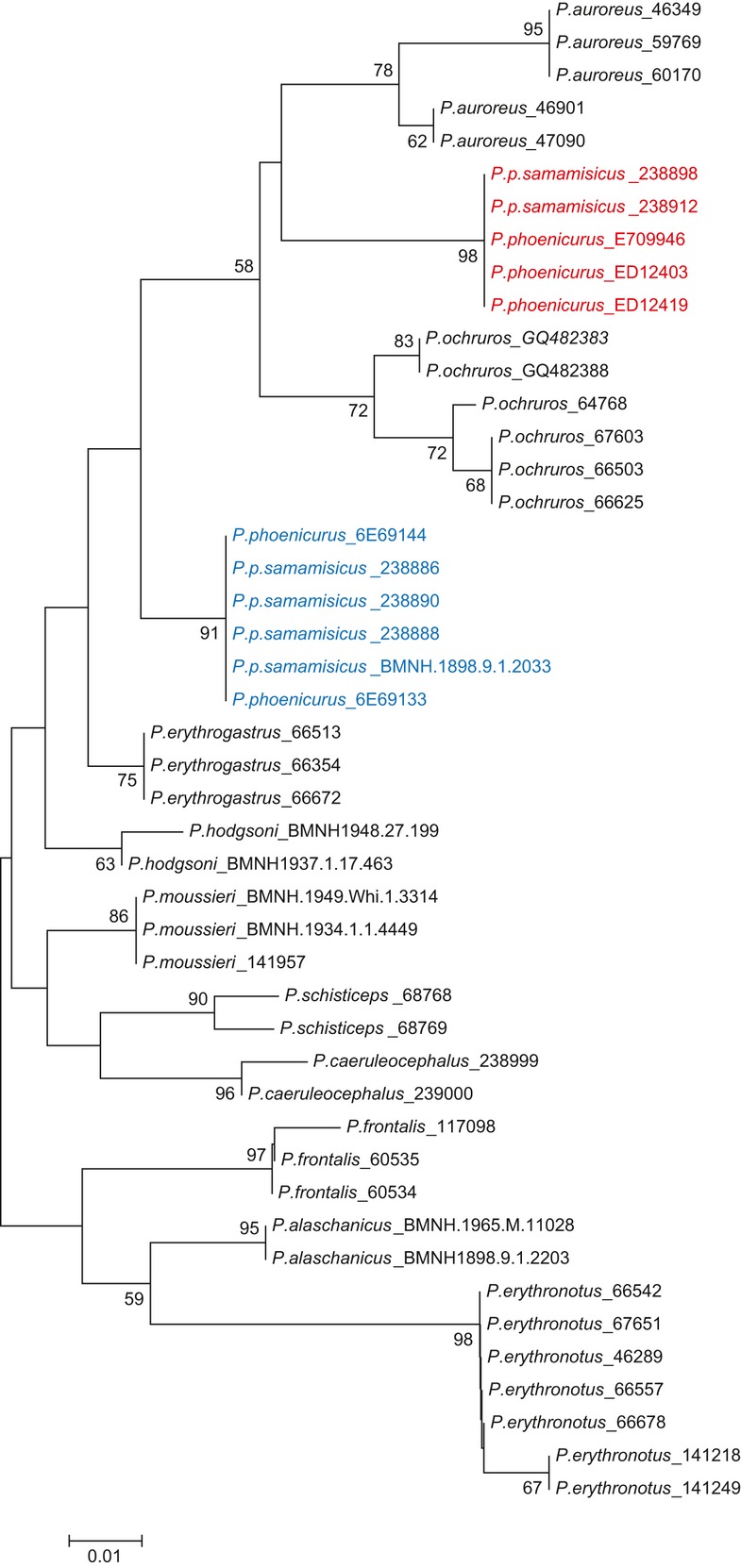
Neighbor-joining tree (K2P, 10,000 bootstrap replicates), based on a short fragment of COI (120bp) for all *Phoenicurus* species. Only bootstrap values above 50% are shown. Blue = haplogroup 1 and red = haplogroup 2.

A NJ tree based on the short COI sequence (120bp) from all 11 *Phoenicurus* species shows that the two haplogroups within the common redstart are unique (Fig. 5). Furthermore, in a dataset of long COI sequences (544bp) from seven species, including all the ones that appear to be most similar to the common redstart in the short COI tree (Fig. 5), the two haplogroups cluster together and are clearly different from the other extant *phoenicurus* species (SI Fig. 2).

### Demographic patterns

**Figure 6:**
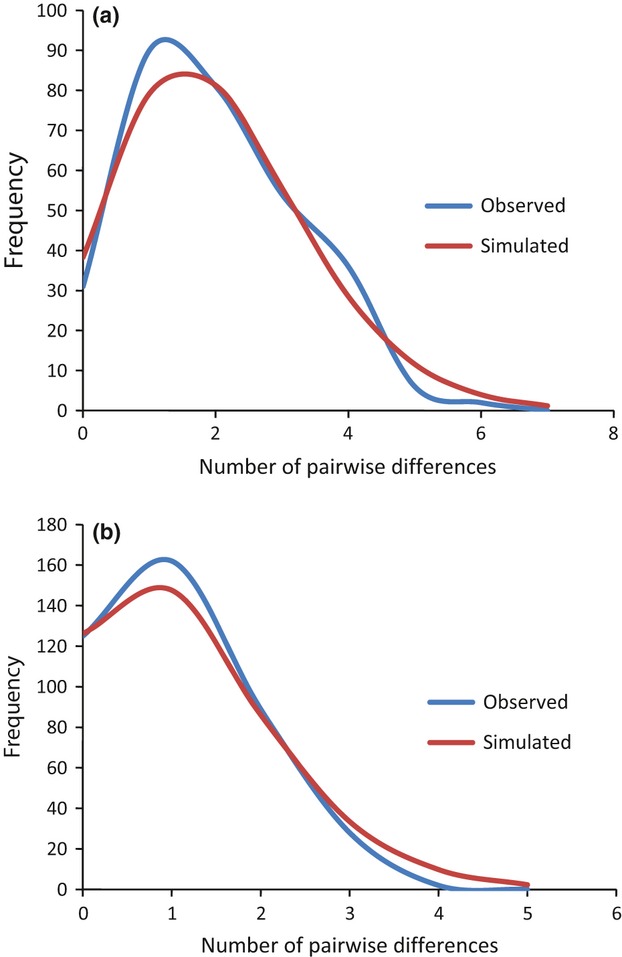
Mismatch distributions based on the combined alignment of the COI and control region for the 54 redstart females from the Norwegian south east population, for (a) haplogroup 1, and (b) haplogroup 2.

Tajima's *D* estimates for the COI and control region combined were significantly negative for both haplogroups (haplogroup 2: Tajima's *D*: −1.855, *P* < 0.05; haplogroup 1: Tajima's *D*: −1.863, *P* < 0.05), and consistent with a population expansion (ArisBrosou and Excoffier 1996). A sudden population expansion was further supported using the mismatch distribution analysis, as both haplogroups fitted this model (Fig. 6a,b). Estimates of the time since the sudden expansion for the two haplogroups, suggest that they both expanded relatively recently, haplogroup 1: *t* = 46,025 years ago (90% CI: 29,040, 65,430), haplogroup 2: *t* = 26 020 years ago (90% CI: 11,357, 40,255).

### Tests of reproductive barriers

**Figure 7:**
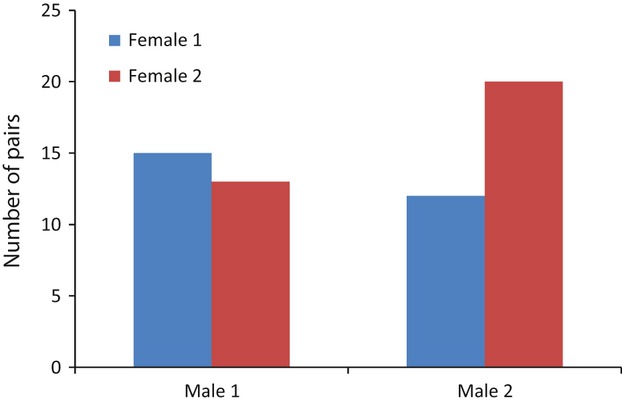
The proportion of common redstart individuals mating with their own, and the opposite haplogroup (*N* = 60 pairs). Blue = haplogroup 1 and red = haplogroup 2.

In the Norwegian population, 35 of 60 pairs consisted of individuals belonging to the same haplogroup (Fig. 7), whereas in the Czech population, four of six sampled pairs consisted of same haplogroup individuals. We found no significant departure from random mating (*N* = 60 pairs, Wald = 1.54, *P* = 0.21) with respect to haplotype. Furthermore, there were no differences in any of the sperm traits between the two haplogroups (Table 4), and no significant differences in any of the sperm traits among the different populations (all F_3,71_ < 0.67, all *P* < 0.57).

**Table 4:** Sperm morphology of males from the two common redstart haplogroups, with the corresponding ANOVA statistics

	Haplogroup 1 (*N* = 43)	Haplogroup 2 (*N* = 32)	ANOVA	
			
Sperm trait	Mean ± SD	Mean ± SD	*F*_1,73_	*P*
Head	17.8 ± 0.9	17.8 ± 0.8	0.04	0.85
Midpiece	129.0 ± 6.0	127.5 ± 5.6	1.58	0.21
Tail	17.4 ± 2.6	17.4 ± 2.7	0.003	0.96
Total	164.4 ± 5.0	162.6 ± 6.6	1.75	0.19
Flagellum	146.6 ± 5.0	144.9 ± 6.5	1.69	0.20

## Discussion

Our results confirm those found in Johnsen et al. (2010), that there are two highly divergent, coexisting mtDNA haplogroups in the common redstart. The two haplogroups show some geographic structure, with haplogroup 1 occurring all over the species distribution, whereas haplogroup 2 occurs predominantly in Northern Europe and parts of Western Asia, an area in which the two lineages are sympatric and interbreed to a large extent. Variation at two Z-linked introns was not related to mtDNA variation. When testing for possible reproductive barriers, we found no evidence for assortative mating and no differentiation in sperm morphology between the two haplogroups.

The magnitude of the divergence within the common redstart (5%) exceeds the divergence found in mtDNA between many sister species (Tavares and Baker 2008). Such deep, sympatric splits have only been found in a handful of other bird species (e.g., Quinn 1992; Webb et al. 2011). There are several possible explanations for the origin and maintenance of such high mitochondrial diversity. First, seemingly high variation in the mtDNA has been shown to sometimes be a result of nuclear mitochondrial pseudogenes, numts (Bensasson et al. 2001). To test for numts, we searched for stop codons and double peaks in the COI region, and double peaks in the control region sequences, and found no evidence for this. These two mtDNA fragments, in addition to 16S sequenced by Johnsen et al. (2010), cover a substantial proportion of the mtDNA, suggesting that the two haplogroups found here are not numts. In addition, Johnsen et al. (2010) ran a XL-PCR, which also supported mitochondrial origin of both haplogroups.

Second, with such a high level of divergence, the two haplogroups might represent cryptic species. However, there was no indication that this is the case, as we found no evidence for assortative mating with respect to haplogroups, no consistent divergence in nuclear introns and no differentiation in sperm morphology. Our results are similar to a study of the common raven by Webb et al. (2011), in which they found a high degree of mixing between two distinct mtDNA clades and no relationship with phenotype. Other studies have found indications of cryptic species based on deep splits in mtDNA, e.g., winter wrens in North America, where song differs and assortative mating was evident (Toews and Irwin 2008). Proponents of DNA barcoding have advocated the use of a threshold level of mtDNA divergence to delimit species (Hebert et al. 2004a,b). Indeed, several recent bird studies using DNA barcoding have suggested provisional species based on such a threshold (<2.5% Kerr et al. 2007, 2009a). Our study shows that using a threshold level to define species may sometimes lead to wrong conclusions, as the common redstart has a divergence of 5%, but is clearly just one biological species. This supports previous critiques of the threshold species concept (Moritz and Cicero 2004). However, as the two haplogroups form a monophyletic group (see SI Fig. 2) that is distinct from other closely related *phoenicurus* species, barcoding can still be used for species identification for this species.

Third, high mtDNA divergence, and inconsistency between mtDNA and nuclear gene trees, can also be a result of hybridization between closely related species, with introgression of mtDNA from one species to the other (Shapiro et al. 2004; Taylor et al. 2011). Common redstarts are known to hybridize with the black redstart in Central Europe, even giving rise to apparently fertile hybrids (Grosch 2004). However, given that none of the two haplogroups matched any of the mtDNA haplotypes found for other extant congenerics, the high divergence in common redstarts seems unlikely to be a result of introgression from other extant *Phoenicurus* species (see also SI Fig. 2). However, we cannot exclude the possibility of introgression of mtDNA from an extinct congeneric or an unsampled extant lineage.

The fourth, and perhaps most parsimonious, explanation of the occurrence of two distinct haplogroups, is that they arose in geographically isolated refugia during previous glaciation periods in Eurasia, and later came into secondary contact. Geographical isolation with secondary contact would predict geographical structure among the mtDNA haplogroups with concomitant differences in demographic history and high nucleotide variation in nuclear introns. The Z-intron nucleotide variation found in this study is the second highest among 22 recently analyzed passerine species (Hogner et al. 2012; Gohli et al., unpubl. data; this study). We found significant genetic differentiation in a subset of seven populations, revealing some geographic structuring, with haplogroup 1 found throughout the whole geographic distribution area, and haplogroup 2 found predominantly in Northwestern Europe and parts of Western Asia (see Fig. 1). On the other hand, the two haplogroups showed similar demographic patterns, with evidence for sudden population expansion during the recent evolutionary history (<50,000 years ago). However, using the conventional molecular clock estimate of 2% divergence per million years (Bromham and Penny 2003; Päckert et al. 2007; but see Pulquério and Nichols 2007; Weir and Schluter 2008), the magnitude of the genetic distance of the haplogroups suggest that they originated in late Pliocene/early Pleistocene (more than 2 million years ago). Thus, the recent glaciation periods in Eurasia might have played a minor role for the origin of the two mtDNA haplogroups observed in the common redstart today. Furthermore, it is difficult to identify the possible location of such old refugia from the present data. The common redstart is taxonomically divided into two subspecies, *P. phoenicurus* and *P. samamisicus*, with *samamisicus* being confined to south-eastern Europe and south-western Asia, including Caucasus, which is a well-known glacial refugium (Hewitt 2000). Hence, the subspecies differentiation possibly reflects a period of geographic isolation, with those evolving into *samamisicus* residing in Caucasus. However, this separation into two subspecies is not related to the divergent mtDNA lineages, as we found a mixture of the two subspecies in the two mtDNA haplogroups (see Fig. 5). This suggests that the isolation event leading to the two divergent mtDNA lineages occurred considerably earlier than the isolation that gave rise to morphological differences between the two subspecies. Alternatively, the mtDNA haplogroups and the subspecies result from the same vicariance event where interbreeding has allowed neutral mtDNA introgression at the same time as selection has maintained the morphological differences.

When two lineages become separated, several factors, including taxon-specific rates of genetic differentiation, the severity of range reduction and timing of allopatric isolation, will play important roles in determining whether these lineages will become reproductively isolated from each other or not (Zamudio and Savage 2003). We found no evidence for reproductive isolation between the haplogroups, neither in the form of assortative mating (a possible precopulatory barrier) or in sperm morphology (a possible postcopulatory, prezygotic barrier; Coyne and Orr 2004). This is similar to the pattern found in the common raven (Webb et al. 2011). Also, we found no difference in sperm morphology between three Norwegian and one Czech population, which is in contrast with other studies showing geographic variation in sperm morphology (Lüpold et al. 2011; Schmoll and Kleven 2011). Furthermore, we found no evidence that nuclear divergence was related to mtDNA divergence. Even if the use of only two Z-linked loci limits the power to detect differences, it should be noted that we used sex-linked loci, which are more often differentiated between young species pairs relative to autosomes (Storchova et al. 2010; Hogner et al. 2012). Hence, if we assume that the divergence in mtDNA is a result of long periods of isolation with secondary contact, our data suggest that the redstart is undergoing speciation in reverse rather than early speciation.

Finally, deep mtDNA divergences may in theory evolve even in the absence of geographic isolation, provided that the effective population size is large enough (Webb et al. 2011). Such coexistence in one panmictic population would predict absence of geographic structure and reproductive barriers, similar mismatch distributions, and lack of divergence in the nuclear introns. We found support for most of these predictions, but the geographic structure in mtDNA and high variation in nuclear introns suggests that this hypothesis cannot fully explain the deep divergence in the common redstart. Possibly, the divergence arose in a period of isolation, for example, during one of the early Pleistocene glacial maxima, and continued to accumulate differences also after secondary contact had been achieved, due to large effective population sizes of both haplogroups. Alternatively, the two mtDNA lineages may have been subjected to differential selection pressures (e.g., local adaptation) that may have accelerated the divergence beyond neutral expectations, as recently suggested in a theoretical study by Irwin (2012).

We conclude that the deep, sympatric mtDNA lineages found in the common redstart do not represent cryptic species, nor are they likely to result from introgression from extant congenerics. Our data suggest that the divergence has evolved in isolated refugia, followed by secondary contact, or represent ancestral lineages that coexist in one panmictic population, or a combination of the two. Discriminating between these alternatives will require deep genetic sampling combined with sophisticated multilocus, coalescence-based analyses. Sympatric mtDNA divergences are relatively rare in birds, but the fact that they occur argues against the use of threshold mtDNA divergences in species delineation.

## Supporting Information

**Table S1.** Detailes of all blood and high quality tissue samples.

**Table S2.** Details of the skin, feather samples and dried blood samples used.

**Table S3.** Details of the sequences downloaded from Genbank.

**Figure S1**. Neighbor-joining tree (K2P, 10,000 bootstrap replicates) based on COI (545bp) for contemporary common redstarts (*N* = 201), with black redstart as outgroup. The two common redstart haplogroups consist of 122 and 79 individuals, respectively. Only bootstrap values above 50% are shown. Blue = haplogroup 1 and red = haplogroup 2.

**Figure S2**. Neighbor-joining tree (K2P, 10,000 bootstrap replicates) based on a long fragment of COI (544bp) for seven *Phoenicurus* species. Only bootstrap values above 50% are shown. Blue = haplogroup 1 and red = haplogroup 2.
