# Supplementary material for: Deep sympatric mitochondrial divergence without reproductive isolation in the common redstart Phoenicurus phoenicurus
Source: Ecol Evol. 2012 Nov 2;2(12):2974–88. doi: 10.1002/ece3.398 (PMC3538993; doi:10.1002/ece3.398)
Supplement: Supplementary file 1 [file ece30002-2974-SD1.docx]

SI Table 1. Detailes of all blood and high quality tissue samples.

| Species | Sex | Date captured | Place captured | Collection/ sampled by | Journal nr | Ring nr | Source | Haplogroup |
| --- | --- | --- | --- | --- | --- | --- | --- | --- |
| *P. p. phoenicurus* | Male | 03.06.2007 | Flåtevatnet Oppland,Norway | NHM, Oslo | 20329 | 5E98118 | Blood | 2 |
| *P. p. phoenicurus* | Male | 14.06.2009 | Flåtevatnet Oppland,Norway | NHM, Oslo | 29527 | 7E44836 | Blood | 1 |
| *P. p. phoenicurus* | Male | 01.05.2009 | Flåtevatnet Oppland,Norway | NHM, Oslo | 29518 | 7E44805 | Blood | 2 |
| *P. p. phoenicurus* | Male | 21.05.2009 | Flåtevatnet Oppland,Norway | NHM, Oslo | 29519 | 7E44813 | Blood | 1 |
| *P. p. phoenicurus* | Male | 29.06.2009 | Flåtevatnet Oppland,Norway | NHM, Oslo | 29529 | 7E44849 | Blood | 1 |
| *P. p. phoenicurus* | Male | 21.05.2009 | Flåtevatnet Oppland,Norway | NHM, Oslo | 29521 | 7E44815 | Blood | 2 |
| *P. p. phoenicurus* | Male | 23.06.2009 | Flåtevatnet Oppland,Norway | NHM, Oslo | 34196 | 7E44835 | Blood | 1 |
| *P. p. phoenicurus* | Male | 01.07.2009 | Flåtevatnet Oppland,Norway | NHM, Oslo | 29531 | 7E44851 | Blood | 2 |
| *P. p. phoenicurus* | Female | 24.06.2007 | Flåtevatnet Oppland,Norway | NHM, Oslo | 20330 | 5E98108 | Blood | 1 |
| *P. p. phoenicurus* | Female | 14.06.2009 | Flåtevatnet Oppland,Norway | NHM, Oslo | 29528 | 7E44837 | Blood | 1 |
| *P. p. phoenicurus* | Female | 21.05.2009 | Flåtevatnet Oppland,Norway | NHM, Oslo | 29520 | 7E44814 | Blood | 1 |
| *P. p. phoenicurus* | Male | 2003 | Kamperud Østfold, Norway | NHM, Oslo | 9811 |  | Blood | 2 |
| *P. p. phoenicurus* | Male | 18.06.2002 | Røros, Norway | NHM, Oslo | 14695 | E770113 | Blood | 2 |
| *P. p. phoenicurus* | Male | 18.06.2002 | Røros, Norway | NHM, Oslo | 14704 | E770122 | Blood | 2 |
| *P. p. phoenicurus* | Male | 17.06.2002 | Røros, Norway | NHM, Oslo | 14713 | E770078 | Blood | 1 |
| *P. p. phoenicurus* | Male | 17.06.2002 | Røros, Norway | NHM, Oslo | 14722 | E770087 | Blood | 2 |
| *P. p. phoenicurus* | Male | 08.06.2009 | Røros, Norway | NHM, Oslo | 37559 | 3E12274 | Blood | 2 |
| *P. p. phoenicurus* | Male | 08.06.2009 | Røros, Norway | NHM, Oslo | 37558 | 3E12273 | Blood | 1 |
| *P. p. phoenicurus* | Male | 09.06.2009 | Røros, Norway | NHM, Oslo | 37562 | 3E12278 | Blood | 1 |
| *P. p. phoenicurus* | Male | 09.06.2009 | Røros, Norway | NHM, Oslo | 37563 | 3E12279 | Blood | 2 |
| *P. p. phoenicurus* | Male | 09.06.2009 | Røros, Norway | NHM, Oslo | 37561 | 3E12277 | Blood | 1 |
| *P. p. phoenicurus* | Male | 08.06.2009 | Røros, Norway | NHM, Oslo | 37560 | 3E12275 | Blood | 1 |
| *P. p. phoenicurus* | Unknown | 08.06.2009 | Røros, Norway | NHM, Oslo | 37555 | 3E12270 | Blood | 2 |
| *P. p. phoenicurus* | Unknown | 08.06.2009 | Røros, Norway | NHM, Oslo | 37554 | 3E12269 | Blood | 1 |
| *P. p. phoenicurus* | Unknown | 08.06.2009 | Røros, Norway | NHM, Oslo | 37553 | 3E12268 | Blood | 1 |
| *P. p. phoenicurus* | Male | 14.06.2006 | Røros, Norway | NHM, Oslo | 25648 |  | Blood | 1 |
| *P. p. phoenicurus* | Male | 22.06.2006 | Røros, Norway | NHM, Oslo | 25657 |  | Blood | 1 |
| *P. p. phoenicurus* | Male | 23.06.2006 | Røros, Norway | NHM, Oslo | 25666 |  | Blood | 1 |
| *P. p. phoenicurus* | Male | 23.06.2006 | Røros, Norway | NHM, Oslo | 25675 |  | Blood | 2 |
| *P. p. phoenicurus* | Male | 23.06.2006 | Røros, Norway | NHM, Oslo | 25685 |  | Blood | 1 |
| *P. p. phoenicurus* | Male | 23.06.2006 | Røros, Norway | NHM, Oslo | 25693 |  | Blood | 2 |
| *P. p. phoenicurus* | Female | 2003 | Kamperud Østfold, Norway | NHM, Oslo | 9812 |  | Blood | 1 |
| *P. p. phoenicurus* | Female | 18.06.2002 | Røros, Norway | NHM, Oslo | 14696 | E770112 | Blood | 1 |
| *P. p. phoenicurus* | Female | 18.06.2002 | Røros, Norway | NHM, Oslo | 14705 | E770121 | Blood | 2 |
| *P. p. phoenicurus* | Female | 17.06.2002 | Røros, Norway | NHM, Oslo | 14714 | E770077 | Blood | 2 |
| *P. p. phoenicurus* | Female | 17.06.2002 | Røros, Norway | NHM, Oslo | 14723 | E770086 | Blood | 2 |
| *P. p. phoenicurus* | Female | 08.06.2009 | Røros, Norway | NHM, Oslo | 37556 | 3E12271 | Blood | 1 |
| *P. p. phoenicurus* | Female | 08.06.2009 | Røros, Norway | NHM, Oslo | 37557 | 3E12272 | Blood | 1 |
| *P. p. phoenicurus* | Female | 09.06.2009 | Røros, Norway | NHM, Oslo | 37564 | 3E12280 | Blood | 2 |
| *P. p. phoenicurus* | Female | 14.06.2006 | Røros, Norway | NHM, Oslo | 25649 |  | Blood | 2 |
| *P. p. phoenicurus* | Female | 22.06.2006 | Røros, Norway | NHM, Oslo | 25658 |  | Blood | 1 |
| *P. p. phoenicurus* | Female | 23.06.2006 | Røros, Norway | NHM, Oslo | 25667 |  | Blood | 2 |
| *P. p. phoenicurus* | Female | 23.06.2006 | Røros, Norway | NHM, Oslo | 25676 |  | Blood | 2 |
| *P. p. phoenicurus* | Female | 23.06.2006 | Røros, Norway | NHM, Oslo | 25686 |  | Blood | 2 |
| *P. p. phoenicurus* | Female | 23.06.2006 | Røros, Norway | NHM, Oslo | 25694 |  | Blood | 2 |
| *P. p. phoenicurus* | Male | 12.06.2009 | Trysil, Norway | NHM, Oslo | 29036 | 3E12874 | Blood | 2 |
| *P. p. phoenicurus* | Male | 12.06.2009 | Trysil, Norway | NHM, Oslo | 29044 | 3E12882 | Blood | 1 |
| *P. p. phoenicurus* | Male | 12.06.2009 | Trysil, Norway | NHM, Oslo | 29045 | 3E12883 | Blood | 2 |
| *P. p. phoenicurus* | Female | 11.06.2009 | Trysil, Norway | NHM, Oslo | 29037 | 3E12875 | Blood | 2 |
| *P. p. phoenicurus* | Female | 12.06.2009 | Trysil, Norway | NHM, Oslo | 29043 | 3E12881 | Blood | 1 |
| *P. p. phoenicurus* | Female | 10.06.2009 | Trysil, Norway | NHM, Oslo | 29030 | 3E12868 | Blood | 2 |
| *P. p. phoenicurus* | Male | 19.06.2002 | Trysil, Norway | NHM, Oslo | 14731 | 2E95422 | Blood | 1 |
| *P. p. phoenicurus* | Male | 20.06.2002 | Trysil, Norway | NHM, Oslo | 14740 | 2E95455 | Blood | 2 |
| *P. p. phoenicurus* | Male | 21.06.2002 | Trysil, Norway | NHM, Oslo | 14748 | 2E95230 | Blood | 2 |
| *P. p. phoenicurus* | Male | 21.06.2002 | Trysil, Norway | NHM, Oslo | 14757 | 2E95463 | Blood | 2 |
| *P. p. phoenicurus* | Male | 21.06.2002 | Trysil, Norway | NHM, Oslo | 14790 | 2E95460 | Blood | 2 |
| *P. p. phoenicurus* | Female | 19.06.2002 | Trysil, Norway | NHM, Oslo | 14732 | 2E95423 | Blood | 1 |
| *P. p. phoenicurus* | Female | 19.06.2002 | Trysil, Norway | NHM, Oslo | 14741 | 2E95430 | Blood | 2 |
| *P. p. phoenicurus* | Female | 21.06.2002 | Trysil, Norway | NHM, Oslo | 14749 | 2E95229 | Blood | 2 |
| *P. p. phoenicurus* | Female | 21.06.2002 | Trysil, Norway | NHM, Oslo | 14758 | 2E95462 | Blood | 2 |
| *P. p. phoenicurus* | Female | 21.06.2002 | Trysil, Norway | NHM, Oslo | 14815 | 2E95461 | Blood | 1 |
| *P. p. phoenicurus* | Male | 11.06.2009 | Trysil, Norway | NHM, Oslo | 29038 | 3E12876 | Blood | 2 |
| *P. p. phoenicurus* | Male | 11.06.2009 | Trysil, Norway | NHM, Oslo | 29035 | 3E12873 | Blood | 1 |
| *P. p. phoenicurus* | Male | 11.06.2009 | Trysil, Norway | NHM, Oslo | 29034 | 3E12872 | Blood | 2 |
| *P. p. phoenicurus* | Male | 11.06.2009 | Trysil, Norway | NHM, Oslo | 29033 | 3E12871 | Blood | 1 |
| *P. p. phoenicurus* | Male | 11.06.2009 | Trysil, Norway | NHM, Oslo | 29032 | 3E12870 | Blood | 2 |
| *P. p. phoenicurus* | Male | 11.06.2009 | Trysil, Norway | NHM, Oslo | 29031 | 3E12869 | Blood | 2 |
| *P. p. phoenicurus* | Male | 10.06.2009 | Trysil, Norway | NHM, Oslo | 37565 | 7E44826 | Blood | 1 |
| *P. p. phoenicurus* | Male | 11.06.2009 | Trysil, Norway | NHM, Oslo | 29039 | 3E12877 | Blood | 2 |
| *P. p. phoenicurus* | Female | 11.06.2006 | Trysil, Norway | NHM, Oslo | 29040 | 3E12878 | Blood | 2 |
| *P. p. phoenicurus* | Male | 11.06.2006 | Trysil, Norway | NHM, Oslo | 29041 | 3E12879 | Blood | 1 |
| *P. p. phoenicurus* | Male | 11.06.2006 | Trysil, Norway | NHM, Oslo | 29042 | 3E12880 | Blood | 1 |
| *P. p. phoenicurus* | Male | 12.06.2006 | Trysil, Norway | NHM, Oslo | 29046 | 3E12884 | Blood | 1 |
| *P. p. phoenicurus* | Male | 14.06.2006 | Trysil, Norway | NHM, Oslo | 25505 | 6E69119 | Blood | 2 |
| *P. p. phoenicurus* | Male | 22.06.2006 | Trysil, Norway | NHM, Oslo | 25521 | 6E69150 | Blood | 2 |
| *P. p. phoenicurus* | Male | 16.06.2006 | Trysil, Norway | NHM, Oslo | 25530 | 6E69147 | Blood | 1 |
| *P. p. phoenicurus* | Male | 14.06.2006 | Trysil, Norway | NHM, Oslo | 25538 | 6E69121 | Blood | 2 |
| *P. p. phoenicurus* | Male | 22.06.2006 | Trysil, Norway | NHM, Oslo | 25543 | 6E69146 | Blood | 2 |
| *P. p. phoenicurus* | Male | 15.06.2006 | Trysil, Norway | NHM, Oslo | 25551 | 6E69138 | Blood | 1 |
| *P. p. phoenicurus* | Male | 15.06.2006 | Trysil, Norway | NHM, Oslo | 25568 | 6E69129 | Blood | 2 |
| *P. p. phoenicurus* | Male | 14.06.2006 | Trysil, Norway | NHM, Oslo | 25591 | 6E69124 | Blood | 2 |
| *P. p. phoenicurus* | Male | 14.06.2006 | Trysil, Norway | NHM, Oslo | 25606 | 6E69128 | Blood | 1 |
| *P. p. phoenicurus* | Male | 16.06.2006 | Trysil, Norway | NHM, Oslo | 25615 | 6E69143 | Blood | 2 |
| *P. p. phoenicurus* | Male | 17.06.2006 | Trysil, Norway | NHM, Oslo | 25623 | 6E69144 | Blood | 1 |
| *P. p. phoenicurus* | Male | 15.06.2006 | Trysil, Norway | NHM, Oslo | 25640 | 6E69132 | Blood | 2 |
| *P. p. phoenicurus* | Male | 19.06.2002 | Trysil, Norway | NHM, Oslo | 14765 | 2E95453 | Blood | 1 |
| *P. p. phoenicurus* | Male | 20.06.2002 | Trysil, Norway | NHM, Oslo | 14773 | 2E95458 | Blood | 1 |
| *P. p. phoenicurus* | Male | 20.06.2002 | Trysil, Norway | NHM, Oslo | 14781 | 2E95456 | Blood | 2 |
| *P. p. phoenicurus* | Male | 20.06.2002 | Trysil, Norway | NHM, Oslo | 14793 | 2E95454 | Blood | 2 |
| *P. p. phoenicurus* | Male | 21.06.2002 | Trysil, Norway | NHM, Oslo | 14789 | 2E95459 | Blood | 1 |
| *P. p. phoenicurus* | Male | 16.06.2006 | Trysil, Norway | NHM, Oslo | 25513 | 6E69140 | Blood | 2 |
| *P. p. phoenicurus* | Male | 15.06.2006 | Trysil, Norway | NHM, Oslo | 25561 | 6E69137 | Blood | 1 |
| *P. p. phoenicurus* | Male | 15.06.2006 | Trysil, Norway | NHM, Oslo | 25577 | 6E69136 | Blood | 1 |
| *P. p. phoenicurus* | Male | 19.06.2006 | Trysil, Norway | NHM, Oslo | 25584 | 6E69145 | Blood | 2 |
| *P. p. phoenicurus* | Male | 15.06.2006 | Trysil, Norway | NHM, Oslo | 25598 | 6E69135 | Blood | 1 |
| *P. p. phoenicurus* | Male | 15.06.2006 | Trysil, Norway | NHM, Oslo | 25632 | 6E69133 | Blood | 1 |
| *P. p. phoenicurus* | Female | 14.06.2006 | Trysil, Norway | NHM, Oslo | 25506 | 6E69118 | Blood | 2 |
| *P. p. phoenicurus* | Female | 22.06.2006 | Trysil, Norway | NHM, Oslo | 25522 | 6E69149 | Blood | 2 |
| *P. p. phoenicurus* | Female | 16.06.2006 | Trysil, Norway | NHM, Oslo | 25531 | 6E69141 | Blood | 1 |
| *P. p. phoenicurus* | Female | 14.06.2006 | Trysil, Norway | NHM, Oslo | 25539 | 6E69120 | Blood | 1 |
| *P. p. phoenicurus* | Female | 16.06.2006 | Trysil, Norway | NHM, Oslo | 25544 | 6E69142 | Blood | 2 |
| *P. p. phoenicurus* | Female | 14.06.2006 | Trysil, Norway | NHM, Oslo | 25552 | 6E69122 | Blood | 2 |
| *P. p. phoenicurus* | Female | 14.06.2006 | Trysil, Norway | NHM, Oslo | 25569 | 6E69123 | Blood | 2 |
| *P. p. phoenicurus* | Female | 14.06.2006 | Trysil, Norway | NHM, Oslo | 25592 | 6E69126 | Blood | 2 |
| *P. p. phoenicurus* | Female | 14.06.2006 | Trysil, Norway | NHM, Oslo | 25607 | 6E69127 | Blood | 2 |
| *P. p. phoenicurus* | Female | 15.06.2006 | Trysil, Norway | NHM, Oslo | 25616 | 6E69130 | Blood | 2 |
| *P. p. phoenicurus* | Female | 15.06.2006 | Trysil, Norway | NHM, Oslo | 25624 | 6E69134 | Blood | 1 |
| *P. p. phoenicurus* | Female | 15.06.2006 | Trysil, Norway | NHM, Oslo | 25641 | 6E69131 | Blood | 2 |
| *P. p. phoenicurus* | Chick | 2002 | Trysil, Norway | NHM, Oslo | 14733 |  | Blood | 1 |
| *P. p. phoenicurus* | Chick | 2002 | Trysil, Norway | NHM, Oslo | 14742 |  | Blood | 1 |
| *P. p. phoenicurus* | Chick | 2002 | Trysil, Norway | NHM, Oslo | 14774 |  | Blood | 2 |
| *P. p. phoenicurus* | Chick | 2002 | Trysil, Norway | NHM, Oslo | 14782 |  | Blood | 1 |
| *P. p. phoenicurus* | Chick | 2002 | Trysil, Norway | NHM, Oslo | 14808 |  | Blood | 2 |
| *P. p. phoenicurus* | Chick | 2006 | Trysil, Norway | NHM, Oslo | 25515 |  | Blood | 2 |
| *P. p. phoenicurus* | Chick | 2006 | Trysil, Norway | NHM, Oslo | 25563 |  | Blood | 2 |
| *P. p. phoenicurus* | Chick | 2006 | Trysil, Norway | NHM, Oslo | 25578 |  | Blood | 1 |
| *P. p. phoenicurus* | Chick | 2006 | Trysil, Norway | NHM, Oslo | 25585 |  | Blood | 1 |
| *P. p. phoenicurus* | Chick | 2006 | Trysil, Norway | NHM, Oslo | 25599 |  | Blood | 2 |
| *P. p. phoenicurus* | Chick | 2006 | Trysil, Norway | NHM, Oslo | 25633 |  | Blood | 1 |
| *P. p. phoenicurus* | Male | 01.07.2009 | Flåtevatnet Oppland,Norway | NHM, Oslo | 29532 | 7E44852 | Blood | 2 |
| *P. p. phoenicurus* | Male | 22.06.2006 | Trysil, Norway | NHM, Oslo | 25521 | 6E69150 | Blood | 2 |
| *P. p. phoenicurus* | Male | 10.05.2007 | Norway | NHM, Oslo | 37552 | 3E12142 | Blood | 2 |
| *P. p. phoenicurus* | Male | 16.06.2008 | Sør-Audal, Oppland,Norway | NHM, Oslo | 24190 | 7E43078 | Blood | 2 |
| *P. p. phoenicurus* | Male | 16.06.2008 | Sør-Audal, Oppland,Norway | NHM, Oslo | 24198 | 7E43079 | Blood | 2 |
| *P. p. phoenicurus* | Male | 16.06.2008 | Sør-Audal, Oppland,Norway | NHM, Oslo | 24205 | 7E43081 | Blood | 1 |
| *P. p. phoenicurus* | Male | 23.05.2008 | Ifrane, Morocco | NHM, Oslo | 24303 |  | Blood | 1 |
| *P. p. phoenicurus* | Male | 02.05.2010 | Hradec Kràlové, Czech Republic | NHM, Oslo | 37494 | TE95873 | Blood | 1 |
| *P. p. phoenicurus* | Male | 03.05.2010 | Hradec Kràlové, Czech Republic | NHM, Oslo | 37495 | TE95875 | Blood | 1 |
| *P. p. phoenicurus* | Male | 03.05.2010 | Hradec Kràlové, Czech Republic | NHM, Oslo | 37496 | TE95876 | Blood | 1 |
| *P. p. phoenicurus* | Male | 04.05.2010 | Hradec Kràlové, Czech Republic | NHM, Oslo | 37471 | TE95879 | Blood | 1 |
| *P. p. phoenicurus* | Male | 04.05.2010 | Hradec Kràlové, Czech Republic | NHM, Oslo | 37497 | TE95880 | Blood | 1 |
| *P. p. phoenicurus* | Male | 05.05.2010 | Hradec Kràlové, Czech Republic | NHM, Oslo | 37453 | TE95881 | Blood | 2 |
| *P. p. phoenicurus* | Male | 21.05.2010 | Hradec Kràlové, Czech Republic | NHM, Oslo | 37461 | TE95889 | Blood | 1 |
| *P. p. phoenicurus* | Male | 04.05.2010 | Hradec Kràlové, Czech Republic | NHM, Oslo | 37469 | TE95877 | Blood | 2 |
| *P. p. phoenicurus* | Male | 02.07.2010 | Hradec Kràlové, Czech Republic | NHM, Oslo | 40509 | TK22632 | Blood | 1 |
| *P. p. phoenicurus* | Male | 29.06.2010 | Hradec Kràlové, Czech Republic | NHM, Oslo | 40508 | TK22631 | Blood | 1 |
| *P. p. phoenicurus* | Male | 23.06.2010 | Hradec Kràlové, Czech Republic | NHM, Oslo | 40505 | TK22627 | Blood | 1 |
| *P. p. phoenicurus* | Female | 04.05.2010 | Hradec Kràlové, Czech Republic | NHM, Oslo | 37472 | TE95878 | Blood | 1 |
| *P. p. phoenicurus* | Male | 03.05.2010 | Hradec Kràlové, Czech Republic | NHM, Oslo | 37452 | TE95874 | Blood | 2 |
| *P. p. phoenicurus* | Female | 21.05.2010 | Hradec Kràlové, Czech Republic | NHM, Oslo | 37460 | TE95888 | Blood | 1 |
| *P. p. phoenicurus* | Female | 27.05.2010 | Hradec Kràlové, Czech Republic | NHM, Oslo | 37470 | TE95897 | Blood | 1 |
| *P. p. phoenicurus* | Female | 25.05.2010 | Hradec Kràlové, Czech Republic | NHM, Oslo | 37487 | TE95890 | Blood | 1 |
| *P. p. phoenicurus* | Female | 24.06.2010 | Hradec Kràlové, Czech Republic | NHM, Oslo | 40507 | TK22630 | Blood | 1 |
| *P. p. phoenicurus* | Female | 24.06.2010 | Hradec Kràlové, Czech Republic | NHM, Oslo | 40506 | TK22629 | Blood | 1 |
| *P. p. phoenicurus* | Female | 23.06.2010 | Hradec Kràlové, Czech Republic | NHM, Oslo | 40504 | TK22626 | Blood | 1 |
| *P. p. phoenicurus* | Female | 21.05.2010 | Hradec Kràlové, Czech Republic | NHM, Oslo | 37453 | TE95887 | Blood | 1 |
| *P. p. phoenicurus* | Female | 05.05.2010 | Hradec Kràlové, Czech Republic | NHM, Oslo | 37485 | TE95883 | Blood | 1 |
| *P. p. phoenicurus* | Female | 21.05.2010 | Hradec Kràlové, Czech Republic | NHM, Oslo | 37460 | TE95888 | Blood | 1 |
| *P. p. phoenicurus* | Female | 10.06.2010 | Flåtevatnet Oppland,Norway | NHM, Oslo | 34184 | ED12453 | Blood | 2 |
| *P. p. phoenicurus* | Female | 16.06.2010 | Flåtevatnet Oppland,Norway | NHM, Oslo | 34187 | E709938 | Blood | 2 |
| *P. p. phoenicurus* | Male | 16.06.2010 | Flåtevatnet Oppland,Norway | NHM, Oslo | 34188 | E709939 | Blood | 2 |
| *P. p. phoenicurus* | Female | 16.06.2010 | Flåtevatnet Oppland,Norway | NHM, Oslo | 34185 | E709936 | Blood | 2 |
| *P. p. phoenicurus* | Male | 08.06.2010 | Flåtevatnet Oppland,Norway | NHM, Oslo | 34181 | E709934 | Blood | 2 |
| *P. p. phoenicurus* | Male | 11.06.2010 | Trysil, Norway | NHM, Oslo | 37380 | ED12420 | Blood | 1 |
| *P. p. phoenicurus* | Female | 10.06.2010 | Trysil, Norway | NHM, Oslo | 37360 | ED12408 | Blood | 2 |
| *P. p. phoenicurus* | Female | 15.06.2010 | Røros, Norway | NHM, Oslo | 37399 | 3E12563 | Blood | 2 |
| *P. p. phoenicurus* | Female | 19.06.2010 | Flåtevatnet Oppland,Norway | NHM, Oslo | 34189 | E709946 | Blood | 2 |
| *P. p. phoenicurus* | Male | 10.06.2010 | Flåtevatnet Oppland,Norway | NHM, Oslo | 34190 | E709947 | Blood | 1 |
| *P. p. phoenicurus* | Male | 10.06.2010 | Trysil, Norway | NHM, Oslo | 37346 | ED12403 | Blood | 2 |
| *P. p. phoenicurus* | Male | 11.06.2010 | Trysil, Norway | NHM, Oslo | 37370 | ED12414 | Blood | 2 |
| *P. p. phoenicurus* | Male | 10.06.2010 | Trysil, Norway | NHM, Oslo | 37368 | ED12412 | Blood | 2 |
| *P. p. phoenicurus* | Male | 11.06.2010 | Trysil, Norway | NHM, Oslo | 37379 | ED12419 | Blood | 2 |
| *P. p. phoenicurus* | Female | 10.06.2010 | Trysil, Norway | NHM, Oslo | 37369 | ED12413 | Blood | 1 |
| *P. p. phoenicurus* | Female | 23.06.2010 | Flåtevatnet Oppland,Norway | NHM, Oslo | 34195 | E709993 | Blood | 2 |
| *P. p. phoenicurus* | Female | 10.06.2010 | Trysil, Norway | NHM, Oslo | 37342 | ED12401 | Blood | 2 |
| *P. p. phoenicurus* | Male | 10.10.2010 | Trysil, Norway | NHM, Oslo | 37343 | ED12402 | Blood | 2 |
| *P. p. phoenicurus* | Male | 08.06.2010 | Flåtevatnet Oppland,Norway | NHM, Oslo | 34180 | E709933 | Blood | 2 |
| *P. p. phoenicurus* | Male | 09.05.2010 | Flåtevatnet Oppland,Norway | NHM, Oslo | 34162 | E709915 | Blood | 1 |
| *P. p. phoenicurus* | Male | 07.05.2010 | Flåtevatnet Oppland,Norway | NHM, Oslo | 34176 | E709930 | Blood | 1 |
| *P. p. phoenicurus* | Male | 07.06.2010 | Flåtevatnet Oppland,Norway | NHM, Oslo | 34178 | E709931 | Blood | 1 |
| *P. p. phoenicurus* | Female | 23.05.2010 | Flåtevatnet Oppland,Norway | NHM, Oslo | 34193 | E709973 | Blood | 1 |
| *P. p. phoenicurus* | Female | 30.05.2010 | Flåtevatnet Oppland,Norway | NHM, Oslo | 20354 | 5E98117 | Blood | 1 |
| *P. p. phoenicurus* | Female | 08.06.2010 | Flåtevatnet Oppland,Norway | NHM, Oslo | 34179 | E709932 | Blood | 1 |
| *P. p. phoenicurus* | Female | 16.06.2010 | Flåtevatnet Oppland,Norway | NHM, Oslo | 34186 | E709937 | Blood | 1 |
| *P. p. phoenicurus* | Chick | 23.05.2010 | Flåtevatnet Oppland,Norway | NHM, Oslo | 34194 | E709987 | Blood | 2 |
| *P. p. phoenicurus* | Female | 15.06.2010 | Røros, Norway | NHM, Oslo | 37419 | 7E44014 | Blood | 1 |
| *P. p. phoenicurus* | Female | 15.06.2010 | Røros, Norway | NHM, Oslo | 37420 | 7E44015 | Blood | 2 |
| *P. p. phoenicurus* | Female | 15.06.2010 | Røros, Norway | NHM, Oslo | 37421 | 7E44016 | Blood | 1 |
| *P. p. phoenicurus* | Female | 15.06.2010 | Røros, Norway | NHM, Oslo | 37422 | 7E44017 | Blood | 1 |
| *P. p. phoenicurus* | Female | 15.06.2010 | Røros, Norway | NHM, Oslo | 37423 | 7E44018 | Blood | 1 |
| *P. p. phoenicurus* | Male | 15.06.2010 | Røros, Norway | NHM, Oslo | 37397 | 3E12562 | Blood | 1 |
| *P. p. phoenicurus* | Male | 11.06.2010 | Trysil, Norway | NHM, Oslo | 37374 | ED12415 | Blood | 1 |
| *P. p. phoenicurus* | Male | 14.06.2010 | Røros, Norway | NHM, Oslo | 37384 | 3E12558 | Blood | 1 |
| *P. p. phoenicurus* | Male | 16.06.2010 | Røros, Norway | NHM, Oslo | 37407 | 3E12566 | Blood | 1 |
| *P. p. phoenicurus* | Male | 15.06.2010 | Røros, Norway | NHM, Oslo | 37395 | 3E12561 | Blood | 1 |
| *P. p. phoenicurus* | Female | 14.06.2010 | Røros, Norway | NHM, Oslo | 37383 | 3E12557 | Blood | 2 |
| *P. p. phoenicurus* | Male | 11.06.2010 | Trysil, Norway | NHM, Oslo | 37376 | ED12417 | Blood | 2 |
| *P. p. phoenicurus* | Male | 11.06.2010 | Trysil, Norway | NHM, Oslo | 37379 | ED12419 | Blood | 2 |
| *P. p. phoenicurus* | Male | 10.06.2010 | Trysil, Norway | NHM, Oslo | 37364 | ED12411 | Blood | 1 |
| *P. p. phoenicurus* | Male | 11.06.2010 | Trysil, Norway | NHM, Oslo | 37375 | ED12416 | Blood | 1 |
| *P. p. phoenicurus* | Female | 10.06.2010 | Trysil, Norway | NHM, Oslo | 37347 | ED12404 | Blood | 1 |
| *P. p. phoenicurus* | Female | 10.06.2010 | Trysil, Norway | NHM, Oslo | 37354 | ED12407 | Blood | 1 |
| *P. p. phoenicurus* | Female | 10.06.2010 | Trysil, Norway | NHM, Oslo | 37363 | ED12410 | Blood | 1 |
| *P. p. phoenicurus* | Male | 27.06.2010 | Flåtevatnet Oppland,Norway | NHM, Oslo | 34198 | EC33312 | Blood | 2 |
| *P. p. phoenicurus* | Egg | 22.06.2010 | Trysil, Norway | NHM, Oslo | 40529 |  | Egg | 1 |
| *P. p. phoenicurus* | Female | 27.06.2010 | Flåtevatnet Oppland,Norway | NHM, Oslo | 34197 | EC33311 | Blood | 1 |
| *P. p. phoenicurus* | Male | 11.06.2010 | Trysil, Norway | NHM, Oslo | 37377 | ED12418 | Blood | 1 |
| *P. p. phoenicurus* | Male | 11.06.2010 | Trysil, Norway | NHM, Oslo | 37381 | ED12421 | Blood | 2 |
| *P. p. phoenicurus* | Male | 22.06.2010 | Trysil, Norway | NHM, Oslo | 37425 | ED12423 | Blood | 1 |
| *P. p. phoenicurus* | Male | 22.06.2010 | Trysil, Norway | NHM, Oslo | 37426 | ED12424 | Blood | 1 |
| *P. p. phoenicurus* | Male | 22.06.2010 | Trysil, Norway | NHM, Oslo | 37428 | ED12426 | Blood | 1 |
| *P. p. phoenicurus* | Female | 17.06.2002 | Røros, Norway | NHM, Oslo | 14864 | E770072 | Blood | 2 |
| *P. p. phoenicurus* | Female | 10.06.2010 | Trysil, Norway | NHM, Oslo | 14807 | ED12452 | Blood | 2 |
| *P. p. phoenicurus* | Female | 17.06.2002 | Røros, Norway | NHM, Oslo | 14865 | E770073 | Blood | 2 |
| *P. p. phoenicurus* | Female | 18.06.2002 | Røros, Norway | NHM, Oslo | 14840 | E770103 | Blood | 2 |
| *P. p. phoenicurus* | Female | 10.06.2010 | Trysil, Norway | NHM, Oslo | 37349 | ED12405 | Blood | 2 |
| *P. p. phoenicurus* | Female | 10.06.2010 | Trysil, Norway | NHM, Oslo | 34182 | ED12451 | Blood | 2 |
| *P. p. phoenicurus* | Female | 18.06.2002 | Elverum, Norway | NHM, Oslo | 14791 | 1E98294 | Blood | 2 |
| *P. p. phoenicurus* | Female | 17.06.2002 | Røros, Norway | NHM, Oslo | 14871 | E770076 | Blood | 2 |
| *P. p. phoenicurus* | Female | 19.06.2002 | Trysil, Norway | NHM, Oslo | 14800 | 2E95438 | Blood | 1 |
| *P. p. phoenicurus* | Female | 18.06.2002 | Røros, Norway | NHM, Oslo | 14848 | E770059 | Blood | 1 |
| *P. p. phoenicurus* | Female | 10.06.2010 | Trysil, Norway | NHM, Oslo | 14792 | 2E95451 | Blood | 1 |
| *P. p. phoenicurus* | Female | 17.06.2002 | Røros, Norway | NHM, Oslo | 14856 | E770071 | Blood | 1 |
| *P. p. phoenicurus* | Female | 18.06.2002 | Røros, Norway | NHM, Oslo | 14839 | E770095 | Blood | 1 |
| *P. p. phoenicurus* | Female | 18.06.2002 | Røros, Norway | NHM, Oslo | 14841 | E770120 | Blood | 1 |
| *P. p. phoenicurus* | Female | 22.06.2010 | Trysil, Norway | NHM, Oslo | 37427 | ED12425 | Blood | 1 |
| *P. p. phoenicurus* | Male | 03.06.2010 | Dovre, Norway | NHM, Oslo | 40512 | 9E91349 | Blood | 2 |
| *P. p. phoenicurus* | Unknown | 02.08.2010 | Dovre, Norway | NHM, Oslo | 40517 | 9E91513 | Blood | 1 |
| *P. p. phoenicurus* | Female | 09.08.2010 | Dovre, Norway | NHM, Oslo | 40521 | 9E91593 | Blood | 1 |
| *P. p. phoenicurus* | Female | 24.06.2010 | Dovre, Norway | NHM, Oslo | 40516 | 9E91471 | Blood | 2 |
| *P. p. phoenicurus* | Unknown | 05.08.2010 | Dovre, Norway | NHM, Oslo | 40520 | 9E91547 | Blood | 1 |
| *P. p. samamisicus* | Chick | 06.05.2010 | Antalya, Turkey | NHM, Oslo | 37498 |  | Feather | 1 |
| *P. p. samamisicus* | Chick | 06.05.2010 | Antalya, Turkey | NHM, Oslo | 37499 |  | Feather | 1 |
| *P. p. samamisicus* | Chick | 16.05.2010 | Antalya, Turkey | NHM, Oslo | 37514 |  | Feather | 1 |
| *P. p. samamisicus* | Female | 16.05.2010 | Antalya, Turkey | NHM, Oslo | 37520 |  | Tissue | 1 |
| *P. p. samamisicus* | Male | 16.05.2010 | Antalya, Turkey | NHM, Oslo | 37521 |  | Tissue | 1 |
| *P. p. samamisicus* | Female | 17.05.2010 | Antalya, Turkey | NHM, Oslo | 37529 |  | Tissue | 1 |
| *P. p. samamisicus* | Male | 17.05.2010 | Antalya, Turkey | NHM, Oslo | 37530 |  | Tissue | 1 |
| *P. p. samamisicus* | Chick | 06.06.2010 | Antalya, Turkey | NHM, Oslo | 37537 |  | Feather | 1 |
| *P. p. samamisicus* | Male | 24.05.2010 | Antalya, Turkey | NHM, Oslo | 37542 |  | Tissue | 1 |
| *P. p. samamisicus* | Chick | 06.06.2010 | Antalya, Turkey | NHM, Oslo | 37536 |  | Feather | 1 |
| *P. p. samamisicus* | Male | 06.05.2010 | Antalya, Turkey | NHM, Oslo | 37511 |  | Blood | 1 |
| *P. p. samamisicus* | Female | 06.05.2010 | Antalya, Turkey | NHM, Oslo | 37510 |  | Blood | 1 |
| *P. p. samamisicus* | Female | 06.05.2010 | Antalya, Turkey | NHM, Oslo | 37504 |  | Blood | 1 |
| *P. p. samamisicus* | Male | 06.05.2010 | Antalya, Turkey | NHM, Oslo | 37512 |  | Blood | 1 |
| *P. p. samamisicus* | Male | 06.05.2010 | Antalya, Turkey | NHM, Oslo | 37513 |  | Blood | 1 |
| *P. p. samamisicus* | Male | 08.06.2010 | Mazichal, Iran | NHM, Oslo | 38644 |  | Blood | 1 |
| *P. p. samamisicus* | Male | 03.06.2010 | Chika, Jahan Nama, Iran | NHM, Oslo | 38643 |  | Blood | 1 |
| *P. p. samamisicus* | Male | 02.06.2010 | Chika, Jahan Nama, Iran | NHM, Oslo | 38642 |  | Blood | 1 |
| *P. p. samamisicus* | Female | 01.06.2010 | Chika, Jahan Nama, Iran | NHM, Oslo | 38640 |  | Blood | 1 |
| *P. p. samamisicus* | Male | 08.06.2010 | Mazichal, Iran | NHM, Oslo | 38645 |  | Blood | 1 |
| *P. p. samamisicus* | Male | 08.06.2010 | Mazichal, Iran | NHM, Oslo | 38646 |  | Blood | 1 |
| *P. p. samamisicus* | Male | 08.06.2010 | Mazichal, Iran | NHM, Oslo | 38647 |  | Blood | 1 |
| *P. p. samamisicus* | Male | 08.06.2010 | Mazichal, Iran | NHM, Oslo | 38648 |  | Blood | 1 |
| *P. p. samamisicus* | Male | 08.06.2010 | Mazichal, Iran | NHM, Oslo | 38649 |  | Blood | 1 |
| *P. p. samamisicus* | Male | 08.06.2010 | Mazichal, Iran | NHM, Oslo | 38650 |  | Blood | 1 |
| *P. p. samamisicus* | Female | 08.06.2010 | Mazichal, Iran | NHM, Oslo | 38654 |  | Blood | 1 |
| *P. p. samamisicus* | Male | 08.06.2010 | Mazichal, Iran | NHM, Oslo | 38658 |  | Blood | 1 |
| *P. p. samamisicus* | Male | 08.06.2010 | Mazichal, Iran | NHM, Oslo | 38653 |  | Blood | 1 |
| *P. p. samamisicus* | Male | 08.06.2010 | Mazichal, Iran | NHM, Oslo | 38651 |  | Blood | 2 |
| *P. p. samamisicus* | Female | 08.06.2010 | Mazichal, Iran | NHM, Oslo | 38652 |  | Blood | 2 |
| *P. p. samamisicus* | Female | 08.06.2010 | Mazichal, Iran | NHM, Oslo | 38656 |  | Blood | 2 |
| *P. p. samamisicus* | Male | 08.06.2010 | Mazichal, Iran | NHM, Oslo | 38655 |  | Blood | 2 |
| *P. p. samamisicus* | Male | 08.06.2010 | Mazichal, Iran | NHM, Oslo | 38657 |  | Blood | 2 |
| *P. p. phoenicurus* | Male | 12.05.2008 | Valjevo, Serbia | YPM, New Haven | 84332 |  | Tissue | 1 |
| *P. p. phoenicurus* | Male | 12.05.2008 | Valjevo, Serbia | YPM, New Haven | 84337 |  | Tissue | 1 |
| *P. p. phoenicurus* | Female | 12.05.2008 | Valjevo, Serbia | YPM, New Haven | 84342 |  | Tissue | 2 |
| *P. p. phoenicurus* | Male | 09.05.2008 | Valjevo, Serbia | YPM, New Haven | 84284 |  | Tissue | 2 |
| *P. p. phoenicurus* | Female | 09.05.2008 | Valjevo, Serbia | YPM, New Haven | 84301 |  | Tissue | 2 |
| *P. p. phoenicurus* | Unknown | 09.05.2008 | Valjevo, Serbia | YPM, New Haven | 84302 |  | Tissue | 1 |
| *P. p. phoenicurus* | Unknown | 09.05.2008 | Valjevo, Serbia | YPM, New Haven | 84303 |  | Tissue | 1 |
| *P. p. phoenicurus* | Female | 15.05.2005 | Krasnodarskiy Kray, Russia | YPM, New Haven | 140371 |  | Tissue | 1 |
| *P. p. phoenicurus* | Female | 15.05.2005 | Krasnodarskiy Kray, Russia | YPM, New Haven | 140372 |  | Tissue | 1 |
| *P. p. phoenicurus* | Female | 15.05.2005 | Krasnodarskiy Kray, Russia | YPM, New Haven | 140373 |  | Tissue | 1 |
| *P. p. phoenicurus* | Female | 21.05.2005 | Krasnodarskiy Kray, Russia | YPM, New Haven | 140803 |  | Tissue | 1 |
| *P. p. phoenicurus* | Male | 21.05.2005 | Krasnodarskiy Kray, Russia | YPM, New Haven | 140804 |  | Tissue | 2 |
| *P. p. phoenicurus* | Male | 28.05.2004 | Krasnodarskiy Kray, Russia | YPM, New Haven | 140824 |  | Tissue | 1 |
| *P. p. phoenicurus* | Female | 28.05.2004 | Krasnodarskiy Kray, Russia | YPM, New Haven | 140827 |  | Tissue | 1 |
| *P. p. phoenicurus* | Male | 29.05.2004 | Krasnodarskiy Kray, Russia | YPM, New Haven | 140730 |  | Tissue | 1 |
| *P. p. phoenicurus* | Male | 30.05.2004 | Krasnodarskiy Kray, Russia | YPM, New Haven | 140837 |  | Tissue | 1 |
| *P. p. phoenicurus* | Male | 19.06.2006 | Krasnodarskiy Kray, Russia | YPM, New Haven | 101876 |  | Tissue | 1 |
| *P. p. phoenicurus* | Female | 23.06.2006 | Krasnodarskiy Kray, Russia | YPM, New Haven | 101813 |  | Tissue | 1 |
| *P. p. phoenicurus* | Male | 23.06.2006 | Krasnodarskiy Kray, Russia | YPM, New Haven | 101905 |  | Tissue | 1 |
| *P. p. phoenicurus* | Female | 14.06.2004 | Republic of Adygea, Russia | YPM, New Haven | 141204 |  | Tissue | 1 |
| *P. p. phoenicurus* | Male | 15.06.2004 | Republic of Adygea, Russia | YPM, New Haven | 140880 |  | Tissue | 2 |
| *P. p. phoenicurus* | Male | 15.06.2004 | Republic of Adygea, Russia | YPM, New Haven | 141206 |  | Tissue | 1 |
| *P. p. phoenicurus* | Male | 15.06.2004 | Republic of Adygea, Russia | YPM, New Haven | 141207 |  | Tissue | 2 |
| *P. p. phoenicurus* | Male | 05.05.2012 | Viladrau, Spain | NHM, Oslo | 38969 | HH2992 | Blood | 2 |
| *P. p. phoenicurus* | Male | 05.05.2012 | Viladrau, Spain | NHM, Oslo | 68970 | HH2995 | Blood | 1 |
| *P. p. phoenicurus* | Male | 05.05.2012 | Viladrau, Spain | NHM, Oslo | 38971 | HH3000 | Blood | 2 |
| *P. p. phoenicurus* | Male | 19.05.2012 | Viladrau, Spain | NHM, Oslo | 38972 | KH7974 | Blood | 1 |
| *P. p. phoenicurus* | Female | 02.06.2012 | Viladrau, Spain | NHM, Oslo | 38973 | KH7975 | Blood | 2 |
| *P. p. phoenicurus* | Male | 02.06.2012 | Viladrau, Spain | NHM, Oslo | 38974 | KH7976 | Blood | 2 |
| *P. p. samamisicus* | Male | 22.04.2008 | Eilat, Israel | NHM, Oslo | 23855 |  | Blood | 1 |
| *P. p. samamisicus* | Female | 17.09.2007 | Eilat, Israel | NHM, Oslo | 22681 |  | Blood | 2 |
| *P. p. samamisicus* | Male | 22.04.2008 | Eilat, Israel | NHM, Oslo | 23854 |  | Blood | 1 |
| *P. p. samamisicus* | Male | 21.04.2008 | Eilat, Israel | NHM, Oslo | 23850 |  | Blood | 1 |
| *P. p. samamisicus* | Male | 18.04.2008 | Eilat, Israel | NHM, Oslo | 23852 |  | Blood | 1 |
| *P. p. samamisicus* | Male | 16.10.2007 | Eilat, Israel | NHM, Oslo | 22689 |  | Blood | 1 |
| *P. p. samamisicus* | Female | 09.10.2007 | Eilat, Israel | NHM, Oslo | 22683 |  | Blood | 1 |
| *P. p. samamisicus* | Female | 12.10.2007 | Eilat, Israel | NHM, Oslo | 22687 |  | Blood | 1 |
| *P. p. samamisicus* | Unknown | 27.09.2007 | Eilat, Israel | NHM, Oslo | 22682 |  | Blood | 1 |
| *P. p. samamisicus* | Male | 18.04.2008 | Eilat, Israel | NHM, Oslo | 23851 |  | Blood | 1 |
| *P. p. samamisicus* | Male | 12.10.2007 | Eilat, Israel | NHM, Oslo | 22686 |  | Blood | 1 |
| *P. p. samamisicus* | Male | 10.10.2007 | Eilat, Israel | NHM, Oslo | 22685 |  | Blood | 1 |
| *P. p. samamisicus* | Male | 10.10.2007 | Eilat, Israel | NHM, Oslo | 22684 |  | Blood | 1 |
| *P. p. samamisicus* | Female | 16.10.2007 | Eilat, Israel | NHM, Oslo | 22688 |  | Blood | 1 |
| *P. p. phoenicurus* | Male | 02.06.2012 | Viladrau, Spain | NHM, Oslo | 38975 | KH7977 | Blood | 1 |
| *P. p. phoenicurus* | Male | 16.06.2012 | Viladrau, Spain | NHM, Oslo | 38976 | KH7978 | Blood | 2 |
| *P. moussieri* | Unknown | 18.11.2004 | Tunisia | NHM, Copenhagen | 141957 |  | Tissue |  |
| *P.frontalis* | Unknown | 20.06.1990 | Sichuan, China | NHM, Copenhagen | 117098 |  | Tissue |  |
| *P. erythronotus* | Unknown | 21.07.2008 | Tien-Shan mtn, Kyrgyzstan | NHM, Copenhagen | 141249 |  | Tissue |  |
| *P. erythronotus* | Male | 20.07.2008 | Tien-Shan mtn, Kyrgyzstan | NHM, Copenhagen | 141218 |  | Tissue |  |

NHM, Oslo = Natural History Museum, Oslo, Norway; YPM, New Haven = Yale Peabody Museum of Natural History, New Haven, USA; NHM, Copenhagen= Natural History Museum, Copenhagen, Denmark.
