# Supplementary material for: Deep sympatric mitochondrial divergence without reproductive isolation in the common redstart Phoenicurus phoenicurus
Source: Ecol Evol. 2012 Nov 2;2(12):2974–88. doi: 10.1002/ece3.398 (PMC3538993; doi:10.1002/ece3.398)
Supplement: Supplementary file 2 [file ece30002-2974-SD2.docx]

SI Table 2. Details of the skin, feather samples and dried blood samples used

| Species | Sex | Date captured | Place captured | Collection/ sampled by | Journal nr | Ring nr | Source | Haplogroup |
| --- | --- | --- | --- | --- | --- | --- | --- | --- |
| *P. phoenicurus* | Male | 01.07.1908 | Surnadal, Norway | NHM, Oslo | 1505 |  | Skin | 1 |
| *P. phoenicurus* | Male | 15.07.1886 | Vestre Aker, Norway | NHM, Oslo | 1499 |  | Skin | 2 |
| *P. phoenicurus* | Male | 20.07.1866 | Finnmark, Norway | NHM, Oslo | 1495 |  | Skin | 2 |
| *P. phoenicurus* | Female | 26.09.1908 | Jæren, Norway | NHM, Oslo | 1506 |  | Skin | 1 |
| *P. phoenicurus* | Male | 18.06.1966 | Øvre Pasvik, Norway | NHM, Oslo | 8868 |  | Skin | 1 |
| *P. phoenicurus* | Unknown | 29.06.1886 | Porsgrunn, Norway | NHM, Oslo | 1883 |  | Skin | 1 |
| *P. phoenicurus* | Male | 18.06.1966 | Øvre Pasvik, Norway | NHM, Oslo | 8869 |  | Skin | 2 |
| *P. phoenicurus* | Male | 18.06.1966 | Øvre Pasvik, Norway | NHM, Oslo | 8870 |  | Skin | 2 |
| *P. phoenicurus* | Unknown | 22.07.1882 | Drammen, Norway | NHM, Oslo | 1497 |  | Skin | 1 |
| *P. phoenicurus* | Unknown | 10.07.1904 | Gausdal, Norway | NHM, Oslo | 1504 |  | Skin | 1 |
| *P. phoenicurus* | Female | 27.06.1992 | Meltaus area, Finland | Esa Huhta |  | 084393 | Feather | 1 |
| *P. phoenicurus* | Female | 30.06.1994 | Meltaus area, Finland | Esa Huhta |  | 367928 | Feather | 2 |
| *P. phoenicurus* | Female | 26.06.1992 | Meltaus area, Finland | Esa Huhta |  | 084390 | Feather | 2 |
| *P. phoenicurus* | Female | 07.06.1993 | Meltaus area, Finland | Esa Huhta |  | 264871 | Feather | 1 |
| *P. phoenicurus* | Female | 27.06.1992 | Meltaus area, Finland | Esa Huhta |  | 084392 | Feather | 1 |
| *P. phoenicurus* | Female | 04.06.1992 | Meltaus area, Finland | Esa Huhta |  | 264785 | Feather | 2 |
| *P. phoenicurus* | Female | 25.06.1992 | Meltaus area, Finland | Esa Huhta |  | 264759 | Feather | 1 |
| *P. phoenicurus* | Female | 28.06.1993 | Meltaus area, Finland | Esa Huhta |  | 367883 | Feather | 2 |
| *P. phoenicurus* | Female | 16.06.1993 | Meltaus area, Finland | Esa Huhta |  | 367816 | Feather | 1 |
| *P. p. phoenicurus* | Female | 22.06.2006 | North Karelia, Joensuu, Finland | Frode Fossøy |  | r842703 | Blood | 1 |
| *P. p. phoenicurus* | Female | 22.06.2006 | North Karelia, Joensuu, Finland | Frode Fossøy |  | r521699J | Blood | 2 |
| *P. p. phoenicurus* | Female | 19.06.2006 | North Karelia, Joensuu, Finland | Frode Fossøy |  | r521691J | Blood | 2 |
| *P. p. phoenicurus* | Female | 30.06.2006 | North Karelia, Joensuu, Finland | Frode Fossøy |  | r842735J | Blood | 2 |
| *P. p. phoenicurus* | Female | 22.06.2006 | North Karelia, Joensuu, Finland | Frode Fossøy |  | r521700J | Blood | 2 |
| *P. p. phoenicurus* | Female | 09.07.2006 | North Karelia, Joensuu, Finland | Frode Fossøy |  | r842736J | Blood | 1 |
| *P. p. phoenicurus* | Female | 22.06.2006 | North Karelia, Joensuu, Finland | Frode Fossøy |  | r842701J | Blood | 1 |
| *P. p. phoenicurus* | Female | 22.06.2006 | North Karelia, Joensuu, Finland | Frode Fossøy |  | r521692J | Blood | 1 |
| *P. phoenicurus* | Chick | 1999 | Hradec Kràlové, Czech Republic | Jiri Porkert/ NHM Oslo | 37992 |  | Dried blood | 2 |
| *P. phoenicurus* | Chick | 1999 | Hradec Kràlové, Czech Republic | Jiri Porkert/ NHM Oslo | 37993 |  | Dried blood | 1 |
| *P. phoenicurus* | Chick | 1998 | Hradec Kràlové, Czech Republic | Jiri Porkert/ NHM Oslo | 37994 |  | Dried blood | 1 |
| *P. phoenicurus* | Chick | 1998 | Hradec Kràlové, Czech Republic | Jiri Porkert/ NHM Oslo | 37995 |  | Dried blood | 2 |
| *P. phoenicurus* | Chick | 1998 | Hradec Kràlové, Czech Republic | Jiri Porkert/ NHM Oslo | 37996 |  | Dried blood | 1 |
| *P. phoenicurus* | Chick | 1998 | Hradec Kràlové, Czech Republic | Jiri Porkert/ NHM Oslo | 37997 |  | Dried blood | 2 |
| *P. phoenicurus* | Chick | 1998 | Hradec Kràlové, Czech Republic | Jiri Porkert/ NHM Oslo | 37998 |  | Dried blood | 1 |
| *P. phoenicurus* | Chick | 1998 | Hradec Kràlové, Czech Republic | Jiri Porkert/ NHM Oslo | 37999 |  | Dried blood | 1 |
| *P. phoenicurus* | Chick | 1999 | Hradec Kràlové, Czech Republic | Jiri Porkert/ NHM Oslo | 38001 |  | Dried blood | 1 |
| *P. phoenicurus* | Male | 28.05.1929 | Sør-Varanger, Norway | NHM, Copenhagen | 10.725 |  | Skin | 2 |
| *P. phoenicurus* | Male | August, 1930 | Lolland, Denmark | NHM, Copenhagen | 19.839 |  | Skin | 2 |
| *P. phoenicurus* | Male | 15.06.1890 | Denmark | NHM, Copenhagen | 19.845 |  | Skin | 1 |
| *P. phoenicurus* | Male | 25.06.1936 | Fyrskib, Denmark | NHM, Copenhagen | 19.867 |  | Skin | 1 |
| *P. phoenicurus* | Female | 01.09.1905 | Fornas fyr, Denmark | NHM, Copenhagen | 19.869 |  | Skin | 1 |
| *P. phoenicurus* | Female | 01.09.1894 | Lodbjerg fyr, Denmark | NHM, Copenhagen | 19.787 |  | Skin | 1 |
| *P. phoenicurus* | Female | 28.06.1959 | Copenhagen, Denmark | NHM, Copenhagen | 70.991 |  | Skin | 1 |
| *P. phoenicurus* | Unknown | 22.06.1895 | Klampenborg, Denmark | NHM, Copenhagen | 19.877 |  | Skin | 1 |
| *P. phoenicurus* | Male | 07.08.1977 | Sjælland, Denmark | NHM, Copenhagen | 67.349 |  | Skin | 2 |
| *P. phoenicurus* | Male | 09.07.1915 | Lule, Lappmark, Sweden | NHM, Copenhagen | 14.542 |  | Skin | 2 |
| *P. phoenicurus* | Male | 16.07.1915 | Lule, Lappmark, Sweden | NHM, Copenhagen | 4.740 |  | Skin | 1 |
| *P. phoenicurus* | Male | 26.06.1929 | Øvre Passvik, Norway | NHM, Copenhagen | 13.266 |  | Skin | 2 |
| *P. phoenicurus* | Male | 22.09.1909 | Nakkehoved fyr, Denmark | NHM, Copenhagen | 19.795 |  | Skin | 2 |
| *P. phoenicurus* | Male | 10.05.1946 | Copenhagen, Denmark | NHM, Copenhagen | 70.989 |  | Skin | 1 |
| *P. phoenicurus* | Male | 20.05 | Schleswig Holstein, Germany | HZM, London | HZM.83.4468 /199 |  | Skin | 1 |
| *P. phoenicurus* | Male | 03.07.1942 | Westerlane, Kent, England | HZM, London | HZM.76.4461 |  | Skin | 1 |
| *P. phoenicurus* | Male | 01.07.1937 | Bern, Switzerland | HZM, London | HZM.75.4460 |  | Skin | 1 |
| *P. phoenicurus* | Female | 03.07.1942 | Sevenoaks, Kent, England | HZM, London | HZM.61.4446 |  | Skin | 1 |
| *P. phoenicurus* | Male | 17.06.1939 | Bern, Switzerland | HZM, London | HZM.59.4444 |  | Skin | 1 |
| *P. phoenicurus* | Male | 12.05.1951 | Biel, Bern, Switzerland | HZM, London | HZM.46.4431 |  | Skin | 1 |
| *P. phoenicurus* | Male | 07.08.1919 | Kincraig, Scotland | HZM, London | HZM.33.4418 |  | Skin | 1 |
| *P. phoenicurus* | Female | 07.08.1919 | Kincraig, Scotland | HZM, London | HZM.44.4426 |  | Skin | 1 |
| *P. phoenicurus* | Male | 21.07.1952 | Saxony, Germany | HZM, London | HZM.39.4424 |  | Skin | 1 |
| *P. phoenicurus* | Male | 11.05.1937 | Blois, France | HZM, London | HZM.110.18898 |  | Skin | 1 |
| *P. phoenicurus* | Male | 10.07.1934 | Bern, Switzerland | HZM, London | HZM.17.4402 |  | Skin | 1 |
| *P. phoenicurus* | Male | 18.07.1932 | Bern, Switzerland | HZM, London | HZM.11.4396 |  | Skin | 2 |
| *P. phoenicurus* | Male | 13.06.1939 | Bern, Switzerland | HZM, London | HZM.13.4398 |  | Skin | 1 |
| *P. phoenicurus* | Male | 24.07.1953 | Bern, Switzerland | HZM, London | HZM.15.4400 |  | Skin | 1 |
| *P. phoenicurus* | Male | 23.05.1930 | Bern, Switzerland | HZM, London | 136.HZM.64386 |  | Skin | 1 |
| *P. phoenicurus* | Male | 22.05.1936 | Bern, Switzerland | HZM, London | HZM.8.4393 |  | Skin | 1 |
| *P. phoenicurus* | Male | 08.08.1970 | Sweden | HZM, London | HZM.64.4449 |  | Skin | 2 |
| *P. phoenicurus* | Male | 10.07.1933 | Bern, Switzerland | HZM, London | HZM.16.4401 |  | Skin | 1 |
| *P. phoenicurus* | Male | 24.06.1923 | Altai, West-Siberia, Russia | NHM, Copenhagen | 31.918 |  | Skin | 1 |
| *P. phoenicurus* | Male | 14.05.1914 | Tomsk, West-Siberia, Russia | NHM, Copenhagen | 31.906 |  | Skin | 1 |
| *P. phoenicurus* | Male | 29.05.1896 | Tomsk, West-Siberia, Russia | NHM, Copenhagen | 31.903 |  | Skin | 1 |
| *P. phoenicurus* | Male | 04.06.1923 | Altai, West-Siberia, Russia | NHM, Copenhagen | 31.917 |  | Skin | 1 |
| *P. phoenicurus* | Male | 28.05.1923 | Altai, West-Siberia, Russia | NHM, Copenhagen | 31.916 |  | Skin | 1 |
| *P. phoenicurus* | Male | 02.07.1914 | Tomsk, West-Siberia, Russia | NHM, Copenhagen | 31.908 |  | Skin | 1 |
| *P. phoenicurus* | Male | 30.08.1917 | Kainsk, West-Siberia, Russia | NHM, Copenhagen | 31.912 |  | Skin | 1 |
| *P. phoenicurus* | Male | 06.09.1917 | Kainsk, West-Siberia, Russia | NHM, Copenhagen | 31.913 |  | Skin | 1 |
| *P. phoenicurus* | Male | August. 1938 | North of Tunis, Tunisia | NHM, Copenhagen | 28.807 |  | Skin | 1 |
| *P. phoenicurus* | Male | 27.11.1910 | Sørvaag, Faroe Islands | NHM, Copenhagen | 71.590 |  | Skin | 1 |
| *P. phoenicurus* | Male | 01.05.1898 | Sørvaag, Faroe Islands | NHM, Copenhagen | 71.588 |  | Skin | 1 |
| *P. phoenicurus* | Male | 01.05.1898 | Sørvaag, Faroe Islands | NHM, Copenhagen | 71.587 |  | Skin | 1 |
| *P. phoenicurus* | Male | Breeding plumage | Dalmatien prap. Pregl. Croatia | NHM, Copenhagen | 31.902 |  | Skin | 1 |
| *P. phoenicurus* | Male | 22.05.1956 | Cambridge-Shire, England | NHM, Tring, London | 1879.4.5.153 |  | Skin | 1 |
| *P. phoenicurus* | Male | 05.08.1935 | Korab Mountains, Macedonia | NHM, Tring, London | 1936:12:15:37. |  | Skin | 2 |
| *P. phoenicurus* | Male | 02.08.1893 | Telschen, Germany | NHM, Tring, London | 1934.1.1.4398 |  | Skin | 1 |
| *P. phoenicurus* | Male | Breeding plumage | Nalchik Trek District, Caucasus,Russia | NHM, Tring, London | 1902.12.7.32 |  | Skin | 2 |
| *P. phoenicurus* | Male | Breeding plumage | Nalchik Trek District, Caucasus,Russia | NHM, Tring, London | 1902.12.7.33 |  | Skin | 2 |
| *P. phoenicurus* | Male | 21.05.1876 | Kavak, Asia Minor, Turkey | NHM, Tring, London | 1898.9.1.2034 |  | Skin | 1 |
| *P. phoenicurus* | Female | 10.07.1919 | Azrou, Lesser Atlas Central, Morocco | NHM, Tring, London | 1919.12.11.209 |  | Skin | 1 |
| *P. phoenicurus* | Female | 23.05.1933 | Kent, England | HZM, London | HZM.97.4481 |  | Skin | 2 |
| *P. phoenicurus* | Male | 10.08.1915 | Fasnakyle, Scotland | HZM, London | HZM.62.4447/202 |  | Skin | 1 |
| *P.p.samamisicus* | Unknown | 22.07.1940 | Mazandaran, Iran | Field Museum, Chicago | 238886 |  | Skin | 1 |
| *P.p.samamisicus* | Juvenil | 16.08.1940 | Mazandaran, Iran | Field Museum, Chicago | 238888 |  | Skin | 1 |
| *P.p.samamisicus* | Juvenil | 17.08.1940 | Bardu Forest, Khoresan, Iran | Field Museum, Chicago | 238890 |  | Skin | 1 |
| *P.p.samamisicus* | Juvenil | 07.07.1940 | Durud, Luristan, Iran | Field Museum, Chicago | 238898 |  | Skin | 2 |
| *P.p.samamisicus* | Unknown | 27.05.1940 | Durud, Luristan, Iran | Field Museum, Chicago | 238912 |  | Skin | 2 |
| *P.p.samamisicus* | Female | 21.05.1876 | Karaku, Pakistan | NHM, Tring, London | BMNH 1898.9.1.2033 |  | Skin | 1 |
| *P. alaschanicus* | Male | May, 1895 | Southern Kuku-Noor range, China | NHM, Tring, London | BMNH 1965.M.11028 |  | Skin |  |
| *P. alaschanicus* | Male | April, pre 1898 | Chuan Che-Sup, China | NHM, Tring, London | BMNH 1898.9.1.2203 |  | Skin |  |
| *P. hodgsoni* | Male | 05.05.1936 | Chayul Valley, Tibet | NHM, Tring | BMNH 1937.1.17.463 |  | Skin |  |
| *P. hodgsoni* | Male | 03.04.1947 | Kongpo, Tibet | NHM, Tring, London | BMNH 1948.27.199 |  | Skin |  |
| *P.frontalis* | Female | 23.05.1925 | Kashmir, India | Field museum, Chicago | 60534 |  | Skin |  |
| *P. frontalis* | Male | 23.05.1925 | Kashmir, India | Field museum, Chicago | 60535 |  | Skin |  |
| *P. caeruleocephalus* | Male | 17.07.1948 | Uttar Predesh Kumaum, India | Field museum, Chicago | 238999 |  | Skin |  |
| *P. caeruleocephalus* | Male | 20.07.1948 | Uttar Predesh Kumaum, India | Field museum, Chicago | 239000 |  | Skin |  |
| *P. schisticeps* | Juvenile | Unknown | Szechwan, China | Field museum, Chicago | 68768 |  | Skin |  |
| *P. schisticeps* | Female | 17.04.1929 | Szechwan, China | Field museum, Chicago | 68769 |  | Skin |  |
| *P.moussieri* | Male | 16.05.1904 | Near Blidah, Algeria | NHM, Tring, London | BMNH 1934.1.1.4449 |  | Skin |  |
| *P.moussieri* | Male | 18.05.1897 | Amsmiz, South Morocco | NHM, Tring, London | BMNH 1949.Whi.1.3314 |  | Skin |  |

NHM, Oslo = Natural History Museum of Oslo, Norway; NHM Copenhagen = Natural History Museum of Copenhagen, Denmark; HZM, London = Harrison Zoological Museum, London, UK; NHM, Tring, London = Natural History Museum of London, Tring, UK; Field museum, Chicago= Field museum, Chicago, USA
