# Supplementary material for: Deep sympatric mitochondrial divergence without reproductive isolation in the common redstart Phoenicurus phoenicurus
Source: Ecol Evol. 2012 Nov 2;2(12):2974–88. doi: 10.1002/ece3.398 (PMC3538993; doi:10.1002/ece3.398)
Supplement: Supplementary file 3 [file ece30002-2974-SD3.docx]

SI Table 3. Details of the sequences downloaded from Genbank.

| Species | Sex | Date captured | Place captured | Collection/ sampled by | Journal nr | Accession nr |
| --- | --- | --- | --- | --- | --- | --- |
| *P. ochruros* | Male | 07.09.1999 | Krasnoyarsk Territory, Russia | Burke Museum, Washington | 64768 | [GQ482386](http://www.ncbi.nlm.nih.gov/nuccore/GQ482386) |
| *P. ochruros* | Male | 06.08.1999 | Tuva, Russia | Burke Museum, Washington | 67603 | [GQ482385](http://www.ncbi.nlm.nih.gov/nuccore/GQ482385) |
| *P. ochruros* | Male | 20.06.2000 | Tuva, Russia | Burke Museum, Washington | 66503 | GQ482384 |
| *P. ochruros* | Male | 11.06.2000 | Tuva, Russia | Burke Museum, Washington | 66625 | [GQ482387](http://www.ncbi.nlm.nih.gov/nuccore/GQ482387) |
| *P. ochruros* | Female |  | Kaliningradskaya Oblast, Russia | ZMMU |  | [GQ482383](http://www.ncbi.nlm.nih.gov/nuccore/GQ482383) |
| *P. ochruros* | Male |  | Kaliningradskaya Oblast | ZMMU |  | [GQ482388](http://www.ncbi.nlm.nih.gov/nuccore/GQ482388) |
| *P. auroreus* | Male | 12.05.1998 | Dornod Aymag Mongolia | Burke Museum, Washington | 59769 | [GQ482372](http://www.ncbi.nlm.nih.gov/nuccore/GQ482372) |
| *P. auroreus* | Male | 17.06.1993 | Buryatiya Respublika, Russia | Burke Museum, Washington | 46349 | [GQ482374](http://www.ncbi.nlm.nih.gov/nuccore/GQ482374) |
| *P. auroreus* | Male | 16.06.1993 | Khabarovsk Territory, Russia | Burke Museum, Washington | 47090 | [GQ482373](http://www.ncbi.nlm.nih.gov/nuccore/GQ482373) |
| *P. auroreus* | Male | 06.08.1998 | Tov, Mongolia | Burke Museum, Washington | 60170 | \| [GQ482370](http://www.ncbi.nlm.nih.gov/nuccore/GQ482370) \|  \| \| --- \| --- \| |
| *P. auroreus* | Male | 21.06.1993 | Khabarovsk Territory, Russia | Burke Museum, Washington | 46901 | [GQ482371](http://www.ncbi.nlm.nih.gov/nuccore/GQ482371) |
| *P. erythrogastrus* | Male | 22.06.2000 | Tuva, Russia | Burke Museum, Washington | 66354 | [GQ482377](http://www.ncbi.nlm.nih.gov/nuccore/GQ482377) |
| *P. erythrogastrus* | Male | 23.06.2000 | Tuva, Russia | Burke Museum, Washington | 66513 | [GQ482375](http://www.ncbi.nlm.nih.gov/nuccore/GQ482375) |
| *P. erythrogastrus* | Male | 22.06.2000 | Tuva, Russia | Burke Museum, Washington | 66672 | [GQ482376](http://www.ncbi.nlm.nih.gov/nuccore/GQ482376) |
| *P. erythronotus* | Male | 06.05.1993 | Republic of Gorno-Altay, Russia | Burke Museum, Washington | 46289 | \| [GQ482382](http://www.ncbi.nlm.nih.gov/nuccore/GQ482382) \|  \| \| --- \| --- \| |
| *P. erythronotus* | Male | 29.06.1999 | Tuva, Russia | Burke Museum, Washington | 67651 | [GQ482380](http://www.ncbi.nlm.nih.gov/nuccore/GQ482380) |
| *P. erythronotus* | Male | 29.06.1999 | Tuva, Russia | Burke Museum, Washington | 66542 | [GQ482378](http://www.ncbi.nlm.nih.gov/nuccore/GQ482378) |
| *P. erythronotus* | Male | 07.02.2000 | Tuva, Russia | Burke Museum, Washington | 66557 | [GQ482381](http://www.ncbi.nlm.nih.gov/nuccore/GQ482381) |
| *P. erythronotus* | Male | 24.06.2000 | Tuva, Russia | Burke Museum, Washington | 66678 | [GQ482379](http://www.ncbi.nlm.nih.gov/nuccore/GQ482379) |

ZMMU = Zoological Museum of Moscow University, GQ numbers are Genbank accession numbers
